# Loss of p53-DREAM-mediated repression of cell cycle genes as a driver of lymph node metastasis in head and neck cancer

**DOI:** 10.1186/s13073-023-01236-w

**Published:** 2023-11-17

**Authors:** Kevin Brennan, Almudena Espín-Pérez, Serena Chang, Nikita Bedi, Saumyaa Saumyaa, June Ho Shin, Sylvia K. Plevritis, Olivier Gevaert, John B. Sunwoo, Andrew J. Gentles

**Affiliations:** 1 Stanford Center for Biomedical Informatics Research, Department of Medicine, Stanford University, Stanford, CA USA; 2 Department of Otolaryngology – Head and Neck Surgery, Stanford University School of Medicine, Stanford, USA; 3 Department of Biomedical Data Science, Stanford University, Stanford, CA USA; 4 Department of Pathology, Stanford University, Stanford, CA USA

**Keywords:** Head and neck cancer, Lymph node metastasis, Transcriptomics, Meta-analysis, Single-cell RNA-Seq, p53-DREAM pathway, Cell cycle, Cellular plasticity, Tumor microenvironment

## Abstract

**Background:**

The prognosis for patients with head and neck cancer (HNC) is poor and has improved little in recent decades, partially due to lack of therapeutic options. To identify effective therapeutic targets, we sought to identify molecular pathways that drive metastasis and HNC progression, through large-scale systematic analyses of transcriptomic data.

**Methods:**

We performed meta-analysis across 29 gene expression studies including 2074 primary HNC biopsies to identify genes and transcriptional pathways associated with survival and lymph node metastasis (LNM). To understand the biological roles of these genes in HNC, we identified their associated cancer pathways, as well as the cell types that express them within HNC tumor microenvironments, by integrating single-cell RNA-seq and bulk RNA-seq from sorted cell populations.

**Results:**

Patient survival-associated genes were heterogenous and included drivers of diverse tumor biological processes: these included tumor-intrinsic processes such as epithelial dedifferentiation and epithelial to mesenchymal transition, as well as tumor microenvironmental factors such as T cell-mediated immunity and cancer-associated fibroblast activity. Unexpectedly, LNM-associated genes were almost universally associated with epithelial dedifferentiation within malignant cells. Genes negatively associated with LNM consisted of regulators of squamous epithelial differentiation that are expressed within well-differentiated malignant cells, while those positively associated with LNM represented cell cycle regulators that are normally repressed by the p53-DREAM pathway. These pro-LNM genes are overexpressed in proliferating malignant cells of *TP53* mutated and HPV + ve HNCs and are strongly associated with stemness, suggesting that they represent markers of pre-metastatic cancer stem-like cells. LNM-associated genes are deregulated in high-grade oral precancerous lesions, and deregulated further in primary HNCs with advancing tumor grade and deregulated further still in lymph node metastases.

**Conclusions:**

In HNC, patient survival is affected by multiple biological processes and is strongly influenced by the tumor immune and stromal microenvironments. In contrast, LNM appears to be driven primarily by malignant cell plasticity, characterized by epithelial dedifferentiation coupled with EMT-independent proliferation and stemness. Our findings postulate that LNM is initially caused by loss of p53-DREAM-mediated repression of cell cycle genes during early tumorigenesis.

**Supplementary Information:**

The online version contains supplementary material available at 10.1186/s13073-023-01236-w.

## Background

Head and neck cancers (HNCs) arise from squamous epithelial cells within the mucosal linings of the oral cavity, larynx, oropharynx, and hypopharynx. HNC is a leading cause of mortality worldwide, accounting for approximately 4.7 million cancer-related deaths per year [1]. The prognosis of HNC (~ 50% 5-year survival) has remained poor over recent decades [2]; however, there is considerable variability in survival and treatment response between patients. This variability likely reflects the inherent heterogeneity of HNC, which occurs in multiple subanatomic regions and can be caused by different etiological factors [3, 4]. Prognosis is currently assessed using clinical stage at presentation, based on the size and location of the primary tumor, presence of lymph node metastases (LNMs) and distant metastases, and by clinical examination and cytology [5]. LNMs represent an independent prognostic factor and are associated with increased risk of metastasis to distant organs [6–8]. Distant metastases confer dismal prognosis, yet most HNC-related deaths occur without evidence of them [6, 7], in contrast to many other solid tumor types.

Greater understanding of the biological factors that influence prognosis in HNC could enable development of clinical biomarkers to improve risk stratification and could lead to novel targeted therapies. These are needed since most patients either do not respond to standard HNC treatment, which includes a combination of surgery, radiotherapy, and chemotherapy; or develop resistance to it [9]. Only a minor subset of patients responds to immune checkpoint immunotherapies [10, 11] or to the epidermal growth factor receptor (EGFR) inhibitor cetuximab, the only targeted agent currently in use [12, 13].

There is a growing understanding of the pathobiological factors that influence HNC progression. A subset of HNCs that occur within the oropharynx are caused by human papillomavirus (HPV). HPV positive (HPV + ve) oropharyngeal cancer (OPC) is associated with favorable survival and therapeutic response relative to HPV − ve [14, 15]. HPV + ve HNC represents a biologically and clinically distinct entity from HPV − ve HNC, which is primarily caused by smoking and/or alcohol use and is associated with *TP53* mutations in ~ 80% of cases [16–18]. Within HPV − ve HNC, *TP53* mutations are associated with worse survival [16] as well as increased incidence of lymph node [19, 20] and distant metastases [21]. Other prognostic factors include smoking, driver mutations, and molecular pathways, and variability in the cellular composition of the tumor microenvironment (TME) [4, 22–24]. In particular, higher levels of infiltrating CD4 + and CD8 + T lymphocytes are associated with better prognosis [25, 26]. Processes promoting cancer progression and invasiveness have also been reported, including epithelial to mesenchymal transition (EMT), hypoxia, and angiogenesis [4, 27].

Here we sought to identify pathways and cellular processes that drive HNC progression based on analysis of transcriptomic data. Prior studies have reported on genes that are associated with clinical outcomes in HNC; however, these studies provide conflicting results, perhaps due to differences in sample processing, data generation, cohort composition, and inconsistent sample annotation. Here we used a comprehensive meta-analysis approach to identify genes that are robustly associated with two clinical outcomes—LNM status and patient survival. We identified cell types that express these prognostic genes through integration with single-cell and bulk-sorted RNA-seq data, revealing prognostic cell types and cellular processes.

## Methods

### Curating HNC gene expression datasets

HNC gene expression studies were primarily accessed from GEO and ArrayExpress. Relevant studies were identified using the search terms “Cancer” in combination with the terms “Head and neck,” “Oral,” “Laryngeal,” “Oropharyngeal,” and “Hypopharyngeal,” and by reviewing all datasets that were retrieved by these searches. For GEO searches, datasets were restricted to those with a minimum of ten samples. We identified additional datasets by searching the reports that were associated with these datasets as well as additional review articles, until we were unable to identify any additional suitable datasets. Clinical data was accessed from the metadata that accompanied each dataset within databases, as well as from relevant reports. Where data that was needed to perform the survival and LNM meta-analysis was incompletely reported, authors and journals were contacted to request these data.

All clinical metadata related to survival (Any survival measure) and LNM was retrieved. Also retrieved, where available, were data indicating tumor grade. Other variables that were retrieved included demographic information (patient age, sex, and reported ancestry (race or ethnicity)), clinicopathological variables (tumor subsite, HPV status, measure of HPV status), details of the patient study (country or sample collection), and data pertaining to HNC-related risk habits (smoking and alcohol consumption status and intensity measures). For the TCGA study, HPV status data was obtained from a publication that applied VirusScan [28] to detect HPV RNA within raw RNA sequencing reads, representing the most complete source of HPV status data in terms of patient numbers. To spot-check the accuracy of clinical data, patient sex was inferred based on the ratio of expression of the *XIST* and *RPS4Y1* genes and compared with clinical annotation of sex. This resulted in exclusion of two studies that had inconsistent clinical data.

The curated data compendium included a combined total of 2074 primary HNCs derived from 29 studies (Additional file 2: Table S1). Meta-analyses were performed to identify genes associated with patient survival, LNM status, and tumor grade, applied to the subset of HNCs that were annotated for each variable: These included 1638 HNCs (across 16 cohorts) with survival outcome data, 1449 HNCs (20 cohorts) with LNM status data, and 1139 HNCs (13 cohorts) with tumor grade data.

### Processing gene expression data (meta-analysis datasets)

Gene expression datasets that were generated using Affymetrix arrays (*N* = 21) were processed as follows. To ensure accurate annotation of microarray probes, raw data (.CEL files) were accessed and processed using the “affy” R package in combination with platform-specific custom CDF files that were accessed from Brainarray (http://brainarray.mbni.med.umich.edu/). Expression datasets were normalized using the mas5 algorithm. Samples were next restricted to primary tumors, followed by quantile normalization of the expression data. Probe-level data was next summarized to gene-level data using the WGCNA package [29], using the default “maxmean” method for probe filtering. For each gene, this method selects the probe with the maximum mean expression across all samples as a representative measure of the gene. Summarized gene data were log2 transformed and converted to standard gene expression scores. For each gene, standard gene expression scores were calculated for each patient sample by subtracting the mean expression of the gene and dividing by the standard deviation. Statistical pipelines that were used to perform meta-analyses were applied to standard scores.

Eight datasets were generated using non-Affymetrix microarrays (Microarrays that were manufactured by Agilent, Illumina, and the German Cancer Research Center). These datasets were downloaded from GEO as series matrix files using the GEOquery R package. These datasets were preprocessed as follows: Gene names were converted to Entrez IDs using array annotation “Platform” files that accompanied each dataset. Where Entrez IDs were not included in the annotation file, gene names were converted to Entrez IDs using biomaRt [30]. Datasets were restricted to primary tumors and were filtered to remove samples with missing data for 10% or greater of genes, and to remove genes that had missing data for 10% or greater of samples. Datasets were then quantile normalized. For genes with multiple probes, the WGCNA package was used to identify the probe with the maximum mean expression across samples, which was selected to be a representative measure for each gene. Datasets were then log2 transformed if not already in log2 space and converted to standard gene expression scores as described for Affymetrix-based datasets.

Preprocessed TCGA bulk RNA-Seq data (gene-level HTSeq counts) were downloaded from TCGAbiolinks [31]. TCGA data was processed for meta-analyses using an approach that was consistent with array-based datasets: The dataset was restricted to primary tumor samples and then quantile normalized. Gene names were converted from Ensembl IDs to Entrez IDs using biomaRt [32]. Ensembl ID-level data was summarized to Entrez gene-level data using the WGCNA package “CollapseRows” function. The default “maxmean” method was used to select features with higher expression where Entrez IDs matched multiple Ensembl IDs. The datasets were then log2 transformed and converted to standard gene expression scores as described for Affymetrix-based datasets.

For applications other than meta-analyses, TCGA RNA-Seq data was processed using an alternative normalization approach in order to process primary HNC and tumor-adjacent normal samples in parallel, as quantile normalization assumes similar data distributions across samples [33]. HTSeq counts were converted to standard scores such that expression data for each HNC sample had a mean of zero and standard deviation of 1. Standard scores were then log2 transformed and batch corrected (correcting for sample plate) using COMBAT [34]. Gene names were converted from Ensembl IDs to Entrez IDs using biomaRt [30]. Ensembl ID-level data was summarized to Entrez gene-level data using the WGCNA package “CollapseRows” function [29]. The default “maxmean” method was used to select features with higher expression where Entrez IDs matched multiple Ensembl IDs.

### Meta-analysis of genes associated with survival

This meta-analysis included all datasets that had at least 20 primary HNCs with survival and gene expression data (*N* = 16 studies with a combined total of 1638 HNCs). Clinical data pertaining to all measures of survival was accessed for each study, and survival time was converted to months. Survival analysis was performed using overall survival (OS) where possible, and other survival measures (progression-free survival, or distant metastasis-free survival) where OS was not reported (Table 1). For each dataset separately, Cox regression models were used to calculate *z*-scores for association of each gene with survival. For genes that were represented in two or more datasets (*N* = 23,558), Liptak’s weighted meta-z test [35, 36] was used to combine *z*-scores for each dataset into a single “meta-z-score,” a summary statistic that indicates the association of gene with survival across studies. Liptak’s meta-*z* test was applied with weights set to the square roots of dataset sample sizes. Genes were considered to be significantly adversely associated with survival (anti-survival) if they had a meta-*z*-score of 3.09 or greater (i.e., *P* < 0.001) and favorably prognostic (pro-survival) if they had a meta-*z*-score of − 3.09 or less.

**Table 1:** Gene expression studies that were included in meta-analyses to identify survival and lymph node metastasis-associated genes

First author /Study_label^a^	Pubmed reference	Study accession^b^	*N* genes^c^	*N* patients survival (Censored|Event, (% event))^d^	*N* patients LNM (LNM0|LNM + , (% LNM +))^e^
TCGA	25631445	TCGA	18803	299|220 (42)	176|244 (58)
Wichmann [19]	26095926	GSE65858	15820	168|85 (34)	77|176 (70)
Walter [37]	23451093	GSE39366	12546	73|64 (47)	51|66 (56)
Fountzilas [38]	23950933	GSE27020	12265	75|34 (31)	NA
Lohavanichbutr [39]	23319825	GSE41613	20408	46|51 (53)	NA
Jung [40]	23757353	E-MTAB-1328	20408	48|41 (46)	17|63 (79)
Lohavanichbutr [39]	23319825	GSE42743	20408	32|42 (57)	29|45 (61)
Thurlow [41]	20458058	Thurlow	17079	43|20 (32)	30|34 (53)
Pickering [42]	23619168	GSE41116	17284	18|24 (57)	16|26 (62)
Bhosale [43]	28433800	GSE85195	19596	26|5 (16)	22|10 (31)
Chung_GSE3292 [44]	16943533	GSE3292	20408	24|8 (25)	10|21 (68)
García-Escudero [45]	29598951	GSE95805	18203	18|13 (42)	25|6 (19)
Chung_GSE2837 [46]	16912200	GSE2837	17473	14|14 (50)	7|21 (75)
Ambatipudi [47]	28433800	GSE23558	19596	18|9 (33)	14|13 (48)
Reis [48]	21989116	GSE31056	20408	14|9 (39)	NA
Cromer [49]	14676830	GSE2379	8459	7|13 (65)	NA
Pavón [50]	22696598	GSE23036	12265	NA	10|53 (84)
Stansfield [51]	26884679	GSE33205	17284	NA	7|37 (84)
Sticht [52]	18472963	GSE10121	10429	NA	12|21 (64)
Enokida [53]	28977904	GSE78060	20408	NA	5|21 (81)
Ye [54]	18254958	GSE9844	20408	NA	15|11 (42)
O'Donnell [55]	15558013	GSE2280	12265	NA	8|14 (64)
Kuriakose [56]	15170515	GSE6631	8459	NA	13|9 (41)
Toruner [57]	15381369	GSE3524	12265	NA	7|7 (50)

^a^First author/Study_label: Study label consists of first author name combined with gene expression omnibus accession number for studies with the same first author’s name

^b^Study accession: Apart from TCGA, accessions represent gene expression omnibus accession numbers. Platform accession numbers are included for studies with multiple datasets generated using different platforms

^c^N genes: Number of genes in dataset

^d^Number of patients with survival data including patients that were censored and that had an event, as well as the percentage of patient with events

^e^Number of patients with lymph node metastasis (LNM) data including patients that were LNM negative (LNM0) and positive (LNM +), as well as the percentage of LNM + patients

### Meta-analysis of genes associated with LNM

This meta-analysis included all datasets that had at least five LNM + and five LNM0 primary HNCs (*N* = 20 studies with a combined total of 1449 patient primary). LNM data was accessed from reports or metadata files as either LNM status (presence or absence of LNM) or was converted from a continuous measure of LNM burden (LNM stage, ratio, or number of LMs). For each gene that was available in at least half of the studies, the following statistics were calculated for each study separately: The standardized mean difference in expression between LNM + and LNM0 primary HNCs, the standard deviation of expression in each of these groups, and the number of samples in each group. Next, we used random effects models [58] to calculate meta-*z*-scores and effect size summary statistics for the association of each gene with LNM status across studies, based on the combined standardized differences and standard deviations, weighted by study sample size. Genes were considered to be positively associated with LNM (Pro-LNM) if they had a meta-*z*-score of 3.09 or greater (i.e., *P* < 0.001) and negatively associated with LNM (Anti-LNM) if they had a meta-*z*-score of − 3.09 or less.

### Meta-analysis to identify genes associated with tumor grade

A meta-analysis was performed to identify genes that were associated with tumor grade (i.e., level of differentiation), where grade was reported either using a numeric grading system of the level of differentiation upon histological analysis (well, moderate, poor). This meta-analysis consisted of 13 studies with a combined total of 1139 primary HNCs. In each study separately, linear regression was applied to test the association of each gene with grade or differentiation level, treating grade, and differentiation level as ordinal variables. For genes that were represented in two or more datasets (*N* = 25,058 genes), Liptak’s weighted meta-*z* test was used to combine *z*-scores for each dataset into a single “meta-*z*-score,” a summary statistic that indicates the association of gene with grade across studies. Liptak’s meta-z test was applied with weights set to the square roots of dataset sample sizes. Genes were considered to be positively associated with grade (pro-grade) if they had a meta-*z*-score of 3.09 or greater (i.e., *P* < 0.001) and negatively associated with grade (anti-grade) if they had a meta-*z*-score of − 3.09 or less.

### Testing the independence of prognostic gene signatures from HPV status

Regression models were used to test the association of survival gene signatures with survival, adjusted for HPV status, and to test the association of LNM gene signatures with LNM status, adjusted for HPV status. Expression scores were calculated for each prognostic gene signature (i.e., set of prognostic genes) as the mean of expression (standardized gene expression scores) of all genes within the signature. Each patient (primary HNC) was thereby assigned an expression score for each prognostic signature. Survival gene signatures included all genes that were negatively (anti-survival) and positively (pro-survival) associated with survival, as well as genes within survival gene clusters (S1-6). Cox regression models were used to test for association of each survival gene expression score with survival, adjusting for HPV status, in all studies (*N* = 4) that had at least ten patients with complete data for survival, HPV status, and gene expression. LNM gene signatures included all genes that were negatively (anti-LNM) and positively (pro-LNM) associated with LNM, and genes within each LNM gene cluster (L1-6). Logistic regression models were used to test for association of each LNM gene expression score with LNM status, adjusting for HPV status, in all studies (*N* = 6) that had at least ten patients with complete data for LNM status, HPV status, and gene expression. For each prognostic (Survival or LNM) gene signature, an HPV-adjusted meta-*z*-score was calculated using Liptak’s weighted meta-*z* test to combine *z*-scores across studies, weighted by study sample size. Additional analyses performed to investigate effects of HPV status and other potential effect-modifier on gene-survival and gene-LNM associations are described in Additional file 1: Supplementary Methods & Results.

### Gene set enrichment analysis

GSEA was applied to all genes that were analyzed as part of the survival and LNM gene meta-analyses, to identify curated genes that were most significantly associated with each outcome, from a database of 18,993 curated gene sets. GSEA was applied to survival and LNM-associated genes using the “fgsea” R package (bioRxiv. 10.1101/060012). For consistency between survival and LNM-associated genes, genes were ranked by meta-*z*-scores, as this summary statistic was available for both. Curated gene sets were accessed from the Molecular Signatures Database (MSigDB) [59]. Selected for analysis were all gene sets in the “C1,” “C2,” “C5,” “C6,” and “H” gene categories, except for gene sets in the “CGP” (chemical and genetic perturbations) subcategory (*N* = 18,993 gene sets). CGP subcategory gene sets as well as gene sets in other categories (C3, C4, C7, and C8) were excluded due to the sparsity of their annotation, which makes them difficult to interpret. Gene sets with fewer than fifteen or more than 500 gene sets were removed to exclude enrichment that are less statistically and biologically meaningful. GSEA scores and *p*-values were calculated for the p53-DREAM target gene set (A set of 201 p53-DREAM target genes accessed from [60]) by adding this gene set to the list of MSigDB gene sets before performing GSEA.

### Gene set overrepresentation analysis

GSOA was applied to identify gene sets that most significantly overlapped with survival and LNM-gene clusters, from a database of 18,437 curated gene sets. GSOA was performed using the msigdbr package, which was used to access gene sets from MSigDB, in combination with the clusterProfiler package, which was used to perform hypergeometric tests. Selected for analysis were all MSigDB gene sets that were used GSEA (See: *Gene set enrichment analysis*). When applying GSOA to genes derived from each meta-analysis, the background gene list used for GSOA consisted of all genes considered in the meta-analysis, i.e., for which meta-*z*-scores were calculated. These represented genes for which data was available in a sufficient number of studies to be considered.

### Unsupervised clustering of prognostic genes based on co-expression in HNC populations

Phenograph [61], an unsupervised clustering method, was used to cluster survival and LNM-associated genes (That were previously identified by meta-analyses) based on their co-expression within HNC bulk transcriptional data. Phenograph was selected over other unsupervised clustering methods to avoid the step of selecting the number of gene clusters (K) a priori, which is required for other methods and introduces bias. For prognostic genes that were associated with each outcome (survival and LNM), Phenograph was applied to a combined matrix of uniformly processed gene expression profiles from twenty studies. Since Phenograph-based clustering does not tolerate missing data, the gene expression matrices were generated using an approach that maximized the number of prognostic genes and HNC that had complete data. To achieve this, for genes associated with each clinical outcome, studies were restricted to HNCs that included data for at least 80% of genes, and data from these studies was combined into a single matrix. Clustering was then applied to genes that had complete (non-missing) data for all samples within the combined matrix. For survival-associated genes, this approach yielded a combined matrix of 1642 HNCs derived from 20 studies, which had complete data for 958/1212 (79%) of all survival-associated genes. For LNM-associated genes, the combined matrix also included 1642 primary HNCs that were derived from twenty patient studies, which included complete data for 742/877 (85%) of the LNM-associated genes. Phenograph was then applied to the combined matrix of gene expression profiles in order to identify co-expressed gene clusters in the sets of survival and LNM-associated genes. All genes that were represented in the combined gene expression matrices were included in the resulting gene clusters.

### Analysis of periodic expression of LNM-associated genes based on expression in synchronously dividing cells

Data that was previously published by Dominguez et al. [62] was used to investigate cell cycle phase-specific expression of LNM-associated genes. These data were generated by applying bulk RNA-Seq to map transcription in synchronously dividing cells (HeLa) that were collected at fourteen timepoints over the course of two mitotic cycles. Normalized gene expression data in the form of fragments per kilobase of transcript per million mapped reads (FKPM) was accessed from the Dominguez et al. report [62], as was data indicating the cell cycle phase within which each gene was expressed.

### Processing the Stanford scRNA-Seq dataset

This dataset was described in our recent report [63] and is accessible from GEO (Accession number: GSE140042). For the current study, analysis was restricted to primary HNCs that were processed using enzymatic digestion, for consistency with the Puram dataset. Cell Ranger was used to align RNA-Seq reads to the latest GENCODE human transcriptome (Genome build hg38) and to quantify RNA counts. Sparse data matrices were then loaded into a Seurat object, which was filtered to remove genes that were present in ten cells or fewer. Low-quality and dying cells were removed by excluding cells with a unique feature count of fewer than 200 (*N* = 72 cells) as well as cells with a mitochondrial genome fraction of 0.4 or greater. Potential doublets were removed by excluding cells with a unique feature count of greater than 4000 (*n* = 444 cells). Integration was then performed by splitting the dataset into separate Seurat objects, with each object containing all the cells that derived from one HNC sample. Gene expression counts for each cell were normalized using regularized negative binomial regression, and variable genes (*N* = 2000) were found for each sample using the “vst” method [64]. Samples were then integrated into a single gene expression object by finding integration anchors using the “FindIntegrationAnchors” and “IntegrateData” commands. The combined genes were then scaled and centered using linear models. This integration approach removed sample batch effects such that cells clustered by cell type rather than by sample. Unsupervised clustering was applied to the integrated Seurat object in order to identify cell clusters using nearest neighbor modularity optimization [65]. PCA performed with 50 principal components (PCs) and elbow plots were then used to select the appropriate number of PCs. Unsupervised clustering was then applied to cells. To identify the appropriate number of cell clusters (i.e., the appropriate level of granularity), cell clustering was performed at multiple resolutions ranging from 0.3 to 1 in increments of 0.1. The optimal resolution was identified based on visualization of the resulting cell clusters using principal component analysis (PCA) and Manifold Approximation and Projection (UMAP). This approach was used to select the number of clusters that separated the major cell types into different clusters and that separated cell types into subclusters (cell subtypes) only where separate subclusters were clearly visible based on PCA and UMAP visualization. Cell clusters were manually annotated with their cell type and subtype by visualizing their expression of known cell type marker genes. Also visualized were gene expression scores of cell type marker gene signatures that were accessed from PangloaDB [66]. Gene expression scores were calculated for each signature as the mean expression (scaled normalized counts) of all genes in the signature, which indicated the expression of the signature in each cell. Cell type assignments were confirmed by applying Seurat to transfer cluster labels from the preexisting Puram HNC scRNA-Seq dataset [67]. This approach used a model that was trained on primary HNCs from the Puram dataset, for which cell types had been previously annotated, to classify cell clusters in the new Stanford scRNA-Seq dataset. Where multiple cell clusters of the same cell type were observed (such as classic fibroblasts and myofibroblasts), cell subtypes were manually annotated by visualizing gene expression signatures for cell subtypes.

### Processing the Puram scRNA-Seq dataset

The Puram scRNA-Seq dataset was accessed from GEO (Accession number: GSE103322) as a preprocessed series matrix file. The dataset was then loaded into a Seurat object and was split into separate sample objects, with each object containing all of the cells that derived from one sample. Samples were restricted to primary HNCs with a minimum of 200 cells (*N* = 9 samples). The Puram dataset was subsequently processed using Seurat, as described for the Stanford scRNA-Seq dataset (See “Processing the Stanford scRNA-Seq dataset”). Cell type labels that were previously assigned by Puram et al. [67] were accessed from the GEO metadata. The validity of these cell type assignments was confirmed by UMAP-based visualizing expression of cell type marker genes and signatures.

Cells were labeled in a way that was consistent between the Puram and Stanford scRNA-Seq datasets, in order to facilitate comparison between these datasets. For this reason, cells that were labeled as myocytes in the Puram dataset were excluded from all analysis, as cells expressing myocyte markers were not observed in the Stanford dataset. Moreover, while macrophages or dendritic cells were labeled by Puram et al., these cells are labeled as myeloid cells in the current study, as we found that in both the Puram and Stanford scRNA-Seq datasets, myeloid lineage cells clearly separated from other cell types but expressed markers of macrophages, dendritic cells, and monocytes. This is consistent with emerging evidence that cells of the mononuclear phagocyte system (macrophages, dendritic cells, and monocytes) do not represent discrete cell types but have overlapping transcriptional profiles and functions [68, 69].

### Prediction of additional cell phenotypes/states in scRNA-Seq datasets

Cell cycle phase was inferred using Seurat, based on expression of cell phase-specific marker genes. CytoTRACE [70] was applied to the raw count gene expression matrix for all epithelial cells, as per user protocol. CytoTRACE was applied to malignant cells only, according to the user manual recommendation that CytoTRACE be applied separately to cells of different lineages. Epithelial to mesenchymal (EMT) score was calculated as the sum of normalized counts for mesenchymal genes (*VIM*, *CDH2*, *FOXC2*, *SNAI1*, *SNAI2*, *TWIST1*, *GSC*, *FN1*, *ITGB6*, *MMP2*, *MMP3*, *MMP9*, and *SOX10)* minus the sum of normalized counts of epithelial genes (*CDH1*, *DSP*, and *TJP1*), as previously described [71, 72].

### Bulk transcriptional profiling of flow-sorted cells

Bulk RNA-Seq was used to profile transcriptomes of distinct cell populations that were isolated from primary HNCs using fluorescence activated cell sorting (FACS):

#### Patient samples

Primary tumor tissue samples were collected between March 2017 and April 2018 from patients (*n* = 15) undergoing surgical resection of HNSCC (including squamous cell carcinoma of the oral cavity, oropharynx, and larynx) at the Stanford Hospital, Stanford, CA, after informed consent. Inclusion criteria included a diagnosis of HNSCC and age ≥ 18 years. Fresh tumor tissue specimens, with clinical annotation, were collected at the time of extirpative surgery and freshly frozen within 6 h after surgical resection. This study was performed in compliance with ethical regulations outlined in a Stanford Institutional Review Board (IRB)-approved protocol (protocol no. 11402). Details of patient clinicopathologic features are provided in Additional file 2: Table S2.

#### Sample preparation for florescence-activated cell sorting (FACS)

FACS sample preparation included obtaining tumor tissue from consented patients within 4 h after tumor tissue removal. Tumor tissue was placed on ice in 50 μl tissue digestion media, DMEM-F12 + with magnesium and calcium (Corning Cellgro, Manassas, VA), 1%FBS (heat inactivated), 10 units/ml Penicillin-10ug/ml Streptomycin (Gibco, Grand Island, NY), and 25 mM hepes (Gibco, Grand Island, NY). Tumor tissue was thoroughly diced with a sterile scalpel and placed in a gentleMACS C-tube (Miltenyi Biotec, Sunnyvale, CA) containing 1.5 ml of tissue digestion media. Tissue was mechanically digested on the GentleMACS dissociator five times under the human tumor tissue program h_tumor_01. Two milliliters of tissue digestion media and 0.5 ml of 3000U/ml collagenase/1000U/ml hyaluronidase (StemCell Technologies, Vancouver, BC) were added to the C-tube after mechanical digestion. The tissue in the C-tube was incubated at 37° C rotating for 1 h, then filtered with a 40-μm nylon cell strainer (Falcon, Corning, NY) into a 14-ml tube containing 14 ml tissue digestion media and centrifuged at 4 °C for 10 min at 514RCF. The enzymatically digested cell pellet was resuspended in 1–4 ml ACK lysis buffer (Gibco, Grand Island, NY) depending on the pellet size and number of red blood cells, for 2 min on ice. Cells were filtered as before, washed with 14 ml of FACS buffer (phosphate buffered saline) without calcium or magnesium (Corning, Manassas, VA), 2%FBS heat inactivated, 10 units/ml Penicillin-10ug/ml Streptomycin (Gibco, Grand Island, NY), and 1 mM Ultra-pure EDTA (Invitrogen, Carlsbad, CA), and centrifuged at 4 °C for 10 min at 514RCF. Cells were resuspended in FACS buffer, counted on a hemacytometer and washed one more time with FACS buffer. Cells were kept in FACS buffer on ice until flow cytometry staining.

#### Flow cytometry staining and sorting

Cells were incubated in the dark on ice for 30 min with fluorescent markers (Additional file 2: Table S3), at the manufacturers’ recommended concentration, except for DAPI, which was added after the last wash. Cells were washed three times with FACS buffer and sorted on a BD Aria II SORP using the BD FACSDIVA v8.0.1 software into 4 groups, CD3 + CD19 + CD45 + CD68 + (leukocytes), unstained (malignant cells), FAP + (fibroblasts), and CD31 + or CD140a + (endothelial cells) in tissue digestion media containing 30% FBS. Cell sorts had an average efficiency of 86.8% on Purity precision sorting, rerunning sorted samples to test for purity was not performed due to the need for enough RNA to sequence. Cells were spun at 4 °C for 10 min at 514RCF and resuspended in RNAlater stabilization solution (Invitrogen, Carlsbad, CA) at the recommended manufacturer’s concentration and stored at 4 °C for less than a week before RNA extraction.

#### Flow cytometry gating

Cells were analyzed using FlowJo V. 10.6.1 and first gated on single cell size using FSC width and height and cell granularity using SSC width and height (Additional file 1: Figure S1). Live cells were gated using the DAPI stain. From the live cell gate, the leukocyte group in FITC and endothelial group in PE were used to separate out CD3 + CD31 leukocyte cells from CD3 − CD31 endothelial cells. Leukocyte and endothelial negative populations were used to gate further for fibroblasts in APC and the malignant unstained (leukocyte, endothelial, and fibroblast negative) group.

#### Bulk RNA sequencing of flow-sorted cells

RNA was extracted from sorted cells within a week of cell sorting. After washing in PBS, cell pellets were used to prepare RNA with the RNAeasy + micro kit with column removal of genomic RNA. RNA samples were quality controlled using the Agilent 2100 Bioanalyzer system. Library preparation was performed using the SMARTer Stranded Total RNA-seq v2 Pico input mammalian kit (Clontech) at the Stanford Protein And Nucleic acid (PAN) facility. Bulk RNA sequencing was performed using the Illumina Hiseq4000 System, inputting 500 pg–5 ng of total RNA per sample and pooling 8–12 samples into each sequencing lane. Sequencing was performed at the Stanford Center for Genomics and Personalized Medicine (SCGPM) facility. This dataset is accessible from Gene Expression Omnibus (Accession number: GSE113839).

### Preprocessing and analyzing the Stanford flow-sorted cell bulk RNA-Seq data

Trim Galore! was used to perform adaptor trimming and filtering of raw reads. Kallisto [73] was used to align reads to the GENCODE 34 human transcriptome (Genome build hg38). MultiQC was used to perform quality control of RNA-Seq samples based on the output of Trim Galore! and Kallisto. Transcript-level counts were summarized to gene level using tximport [74]. DESeq2 [75] was used to convert gene-level count data (The output of tximport [74]) to a “DESeqDataSeq” object and to normalize the RNA-Seq counts by dividing them by estimated size factors. These normalized RNA-Seq counts were used to identify the cell type that featured highest expression of each prognostic gene, representing the cell type with the maximum mean normalized count value. Normalized counts were log2-transformed prior to data visualization.

### Estimating cell fractions within the Stanford flow-sorted cell bulk RNA-Seq dataset

CIBERSORTx [76] was applied to gene-level transcripts per million (TPM) data in combination with a signature matrix derived from the Puram scRNA-Seq dataset. This signature matrix was derived from HNC scRNA-Seq data, ensuring that the gene signatures used to infer cell fractions were representative of cell types found within HNC tumor microenvironments.

### Preprocessing and analyzing the Huang bulk RNA-Seq dataset

Raw fastq files were accessed from the European Genome Phenome Archive (Dataset ID: EGAD00001004489) and were processed as described for the Stanford bulk RNA-Seq dataset (see “Preprocessing and analyzing the Stanford flow-sorted cell bulk RNA-Seq data”). Wilcoxon rank sum tests were used to test for differences in mean expression (DESeq2-normalized counts) of anti-LNM and pro-LNM genes between primary HNCs and patient-matched LNMs.

### Testing association of gene expression with TP53 mutation status

Association of pro-LNM cluster 4 genes with somatic *TP53* mutations was established based of differential expression analysis within the TCGA [16] and Wichmann [19] bulk gene expression datasets, as well as the Puram scRNA-Seq dataset. Within each bulk gene expression dataset, Wilcoxon rank sum tests were used to test for differences in mean gene expression (normalized counts) of cluster L4 genes between subgroups of HNCs that were stratified based on their *TP53* mutation and HPV status. Cluster L4 gene expression was compared between p53 proficient HNCs (HPV − ve and TP53^wt^) and two separate groups of p53-deficient HNCs, including *TP53*^mut^ (HPV − ve) HNCs, and HPV + ve HNCs. For the TCGA study, *TP53* mutation data was accessed from the MC3 Public MAF file [77]. For the Wichmann study, *TP53* mutation data was accessed from GEO metadata (Accession: GSE65858). Excluded from the analysis were Wichmann study HNCs that were annotated as having “non-disruptive” *TP53* mutations and that were HPV negative, due to the ambiguity of their p53 proficiency. In the Puram scRNA-Seq dataset, multiple linear regression was used to test for association of mean cluster L4 gene expression (Normalized counts) with *TP53* mutation status (the mutation status of the overall tumor), adjusted for cell cycle phase (estimated by Seurat), within malignant cells. *TP53* mutation status (as indicated by targeted or whole exome sequencing) were accessed from the Puram et al. report.

### Analysis of expression of LNM-associated genes in oral premalignant lesions

OPL data was accessed from GEO (Accession: GSE26549) [78]. This dataset included gene expression array data for 86 OPL (oral leukoplakia) biopsies that were annotated with follow-up (oral cancer-free survival) information, 84 of which were also were annotated for histology. Raw expression array. CEL files (Affymetrix Human Gene 1.0 ST Array) were processed using the “affy” R package in combination with a platform-specific custom CDF file that was accessed from Brainarray (http://brainarray.mbni.med.umich.edu/). Expression data were normalized using the mas5 algorithm. Probe-level data was next summarized to gene-level data using the WGCNA package [29], using the default “maxmean” method for probe filtering, and summarized gene data were log2 transformed. Subsequent statistical analyses were applied to log2-transformed data.

### Data analysis software

Data analysis was performed using R versions 3.6.1 and 4.1.0. Bulk RNA-Seq reads were trimmed and filtered using Trim Galore! (Version 0.6.0) and were quality controlled using MultiQC v1.9 within Python 2.7.5. Bulk RNA-Seq reads were pseudoaligned using Kallisto (linux-v0.46.0). Aligned bulk RNA-Seq reads were converted to gene-level estimates using Tximport 1.14.2. Gene-level bulk RNA-Seq counts were normalized using DESeq2 (1.26.0). Single-cell RNA-Seq reads (10x Genomics) were processed and aligned using Cell Ranger (6.1.2). Subsequent processing and analysis of single-cell RNA-Seq data was performed using Seurat (4.1.0). Flow cytometry data were analyzed using FlowJo Cytometry Analysis Software (BD Biosciences). Other programs and tools are indicated in the relevant “Methods” and “Results” sections.

## Results

### Curation of a resource for meta-analysis of HNC gene expression

We assembled a compendium of 29 primary HNC gene expression datasets with accompanying clinical data, representing the largest such resource for HNC. This resource was specifically built to identify genes associated with two outcome variables: patient survival and lymph node metastasis (LNM) status. Meta-analyses were applied to uniformly preprocessed gene expression data, as in our PRECOG resource [79]. Briefly, datasets were quality controlled, normalized, log transformed, and standardized to calculate gene expression profiles. Clinical data were manually curated and included survival and LNM status as well as variables relevant to HNC prognosis, such as tumor grade, tumor subanatomic location, and HPV status. The resulting 29 cleaned studies included 2074 HNC tumors (Additional file 2: Table S1). In total, 1638 patients (across 16 cohorts) had survival outcome data and 1449 patients (20 cohorts) had LNM status (Table 1).

### HNC survival-associated genes reflect TME composition, EMT, and hypoxia

**Fig. 1:**
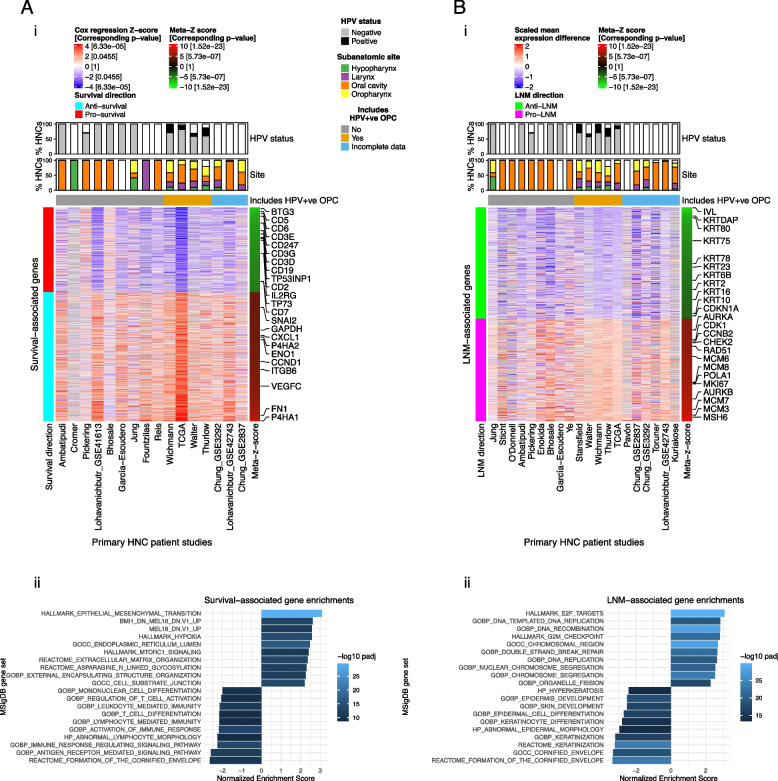
Meta-analysis-based identification of prognostic genes: Genes associated with **A** survival and **B** lymph node metastasis (LNM): *i* Heatmap of cox regression *z*-scores for all survival-associated genes (Rows) in 16 HNC gene expression study datasets (Columns). *Z*-scores indicate the association of genes with survival (overall survival, progression-free survival, or distant metastasis-free survival) within each study. Genes are ordered by meta-*z*-score (Right sidebar), summarizing their association with survival across studies. Bar plots indicate the percentages of HPV positive (HPV + ve) and negative (HPV − ve) cases (HPV status), as well as the percentages of HNCs that occurred within each subanatomic region (Site). White regions of bar plots indicating missing data. Horizontal annotation bars labeled “Includes HPV + ve OPC” indicating studies that include HPV + ve oropharyngeal cancer (OPC) (i.e., studies in which HPV status was a potential confounding factor). “Incomplete data” indicates studies in which clinical annotation was insufficient to determine if the study included HPV + ve OPC. Gene symbol labels highlight functionally significant genes that are mentioned in the main text. *ii* Gene set enrichment analysis (GSEA) of survival-associated genes: Bar plot showing normalized GSEA enrichment scores of gene sets with the strongest positive and negative associations (The top ten of each), out of a total of 18,993 curated gene sets accessed from the Molecular Signatures Database (MSigDB). Depth of color indicates the negative log Benjamini-Hochberg-adjusted *p*-value for enrichment of gene sets. **B** Meta-analysis of genes associated with LNM: *i* Heatmap of scaled mean gene expression differences between primary tumors of LNM + patients and those of LNM0 patients, of all genes that were significantly associated with LNM (rows), in 20 study datasets (columns). Genes are ordered by meta-*z*-score (right sidebar), which summarize their association with LNM across studies. Heatmap annotations are equivalent to those shown for survival-associated genes in **A**. *ii* Bar plot illustrating GSEA of LNM-associated genes. Enrichment scores are shown for the top ten gene sets with the strongest positive and negative associations with LNM

We first identified genes associated with HNC survival across 16 studies. Overall survival (OS) was used where available, while progression-free survival or distant metastasis-free survival was used for other studies (Additional file 2: Table S1). Cox regression models were used to calculate *z*-scores for association of each gene with survival in each dataset. *Z*-scores were then aggregated into a per-gene meta-*z*-score using Liptak’s weighted meta-*z* [35, 36]. Four hundred seventy-nine genes were favorably associated with survival (pro-survival genes; meta-*z* ≤  − 3.09, i.e., *P* < 0.001) and 730 were adversely associated with survival (anti-survival genes; meta-*z* ≥ 3.09) (Fig. 1A and Additional file 2: Table S4). Cox regression *z*-scores were generally consistent between studies that did and did not include HPV + ve OPC (Fig. 1A) and remained significantly associated with survival in a meta-analysis that excluded HPV + ve OPC (Additional file 1: Supplementary Results), indicating that HPV does not drive the association of these genes with survival. Moreover, these genes remained significantly associated with survival after adjustment for age and sex (Additional file 1: Supplementary Results).

We next applied gene set enrichment analysis (GSEA) to identify pathways and functions that were enriched within the survival-associated genes **(**Fig. 1A). GSEA indicated that pro-survival genes were enriched for immune pathways, particularly genes related to antigen receptor-mediated signaling (e.g., *CD247, CD19, IL2RG*) and immune activation (e.g., *CD2*, CD3D/E/G, CD5-7). Anti-survival genes were for epithelial-mesenchymal transition (EMT) (e.g., *SNAI2, ITGB6, FN1*) and hypoxia (e.g., *P4HA1/2, GAPDH, ENO1*), as well as genes regulated by polycomb repressive complex 1 (PRC1) component enzymes BMI and PCGF2 (e.g., *VEGFC*, *CXCL1*, *CCND1*). These enrichments are consistent with known roles for leukocyte infiltration [26, 80] and EMT [81, 82] in protecting against and promoting HNC progression, respectively.

Since survival genes were enriched for multiple oncogenic processes, we next sought to delineate the various prognostic pathways and processes that are represented by these genes. To achieve this, we applied Phenograph [61], an unsupervised clustering method, to cluster the survival-associated genes identified by meta-analysis based on their co-expression within a large HNC population. Phenograph identified six gene clusters: Three (S3, S5-S6) primarily consisted of pro-survival genes, and three (S1-S2, S4) of anti-survival genes (Table 2, Additional file 2: Table S4 and Additional file 1: Figure S2A). Most of these genes had similar *pan-cancer* survival associations when compared to our previous analysis of survival-associated genes (Additional file 1: Figure S3A) [79], indicating that these genes are generally prognostic across cancer types. All survival-associated signatures remained significantly associated with survival in linear models after adjusting for HPV status (Additional file 1: Figure S4A). Gene set overrepresentation analysis (GSOA) (i.e., hypergeometric analysis) was used to identify MSigDB gene sets that most significantly overlapped with each survival gene cluster. Anti-survival cluster S1 was also overrepresented for EMT markers, as well as genes negatively regulated by PRC1, or involved in hypoxia or focal adhesion. Anti-survival cluster S2 included genes involved in ribosome and ribonucleoprotein biogenesis, MTORC1 signaling, and response to protein misfolding. Anti-survival cluster S4 was overrepresented for EMT markers, genes upregulated by TGF beta signaling, and genes encoding extracellular matrix components. Pro-survival gene clusters S5 and S6 were overrepresented for genes related to T cell activation and squamous epithelial differentiation, respectively, but no gene sets significantly overlapped with pro-survival cluster S3.

**Table 2:** Summary of prognostic gene clusters

Prognostic gene cluster	Prognostic association	*N* genes	Overrepresented functions/biological themes^a^	Primary cell (sub)type with highest expression	Association with grade
S1	Anti-survival	240	PRC1 targets, EMT, hypoxia	Ubiquitous (malignant & stroma-skewed)	N/S
S2	Anti-survival	196	MTORC1 signaling, ribosome biogenesis, protein misfolding response	Ubiquitous (malignant-skewed)	N/S
S3	Pro-survival	140	None	Ubiquitous (endothelial & malignant-skewed)	+
S4	Anti-survival	139	EMT, extracellular matrix, TGFB signaling	Fibroblasts	+
S5	Pro-survival	120	Antigen receptor-mediated antitumor immunity	T/NK cells	+
S6	Pro-survival	123	Epithelial differentiation (overlap with cluster L2)	Well-differentiated malignant cells	-
L1	Anti-LNM	162	Epithelial differentiation	Well-differentiated malignant cell cluster	-
L2	Anti-LNM	209	Epithelial differentiation	Well-differentiated malignant cells	-
L3	Anti-LNM	15	Intracellular transport, RNA splicing	Ubiquitous (malignant-skewed)	-
L4	Pro-LNM	196	Cell cycle genes, p53-DREAM targets, DNA replication/repair	G2/M phase/stem-like malignant cells	+
L5	Pro-LNM	146	Regulation of various immune processes, KRAS and IL2-STAT5 signaling	Ubiquitous across non-malignant cell types	+
L6	Pro-LNM	14	RNA splicing	Ubiquitous (malignant-skewed)	+

^a^Identified by gene set overlap analysis (GSOA), detailed in Additional file 2: Table S6

### Epithelial differentiation is a key factor in HNC lymph node metastasis

We next identified genes that are differentially expressed in primary tumors of LNM positive (LNM +) patients relative to primary tumors of LNM negative (LNM0) patients. Random effects models applied to 20 datasets with a total of 1449 primary HNCs identified 420 genes more highly expressed in primary tumors of LNM + patients (pro-LNM genes; meta-*z* ≤  − 3.09) and 457 genes that were lower expressed (anti-LNM genes; meta-*z* ≥ 3.09) (Fig. 1B, Additional file 2: Table S4). Associations of gene signatures with LNM status were independent of HPV status, age, and sex (Fig. 1B, Additional file 1: Figure S4, Supplementary Results).

**Fig. 2:**
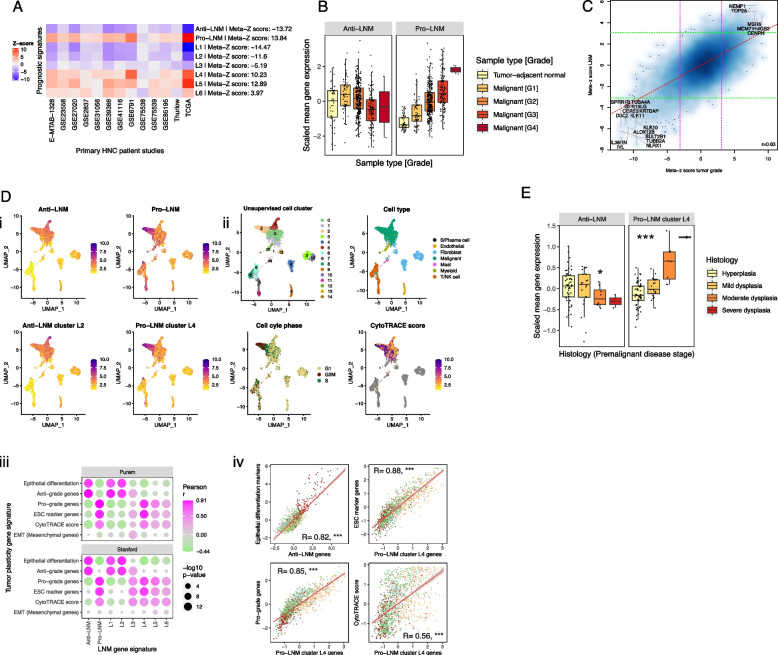
Association of lymph node metastasis (LNM) signatures with epithelial dedifferentiation and dysplasia: **A** Heatmap showing linear regression *z*-scores for association of LNM gene signatures with tumor grade in 13 HNC bulk gene expression studies. *Z*-scores indicate the significance of associations between tumor grade and expression scores of LNM gene signatures within each study. Expression scores were calculated for each LNM gene signature (i.e., set of genes) as the mean of expression of genes within that signature. Row labels indicate meta-*z*-scores for the association of each signature with grade across studies, which were calculated by combining *z*-scores across studies using Liptak’s weighted meta-*z* test. LNM gene signatures include all genes that were negatively (anti-LNM) and positively (pro-LNM) associated with LNM, and genes within LNM gene clusters (L1-6). **B** Box plots showing mean expression of anti-LNM cluster 1 and pro-LNM cluster 4 genes within primary HNCs and tumor-adjacent normal tissue (normal) of the TCGA HNSC study, with HNCs stratified by tumor grade (G1-4). **C** Smoothed scatter plot showing the correlation between meta-*z*-scores for association of genes (points) with tumor grade (*X*-axis) and LNM status (*Y*-axis). Meta-*z*-scores for association of genes with grade and LNM were calculated based on separate meta-analyses. Dashed lines indicate meta-*z*-score significance thresholds (Absolute meta-*z* = 3.09). Regression lines (red dashed line) and Pearson correlation coefficient (r) are indicated. Text labels highlight genes that were among those with the 50 highest and lowest meta-*z*-scores for association with both LNM and grade (i.e., genes that were strongly association with both LNM and grade). **D** ScRNA-Seq analyses indicating the association of LNM signatures with epithelial differentiation and stemness within malignant cells. *i* Uniform Manifold Approximation and Projections (UMAPs) showing expression scores of LNM-associated gene signatures within the Puram scRNA-Seq dataset. Signatures shown include all genes that were negatively (anti-LNM) and positively (pro-LNM) associated with LNM, as well as genes within anti-LNM cluster L2 and pro-LNM cluster L4, the largest unsupervised clusters of LNM-associated genes. *ii* UMAPs corresponding to those shown in *i*, showing cell phenotypes including unsupervised cell cluster, cell type, and cell cycle phase, as well as CytoTRACE score, a measure of transcriptional diversity and stemness [70]. *iii* Heatmap showing correlations of LNM gene signatures with tumor plasticity signatures within the Puram and Stanford scRNA-Seq datasets. Points indicate correlations between expression scores within malignant cells of two scRNA-Seq datasets. Pearson correlation coefficients (*r*) is represented by the point color gradient, while point sizes represent negative log ten *p*-values (linear regression). Expression scores are calculated for each cell as the scaled mean expression (normalized counts) of all genes within a signature (i.e., set of LNM-associated genes). Tumor plasticity gene signatures shown include epithelial differentiation markers: Genes identified as part of an epithelial differentiation-related transcriptional program in HNC (referred to as “Epi dif. 1”) based on the original analysis of the Puram dataset [67], ESC markers: genes specifically expressed in embryonic stem cells (ESCs) [84], tumor grade-associated gene signatures: Genes positively (pro-grade) and negatively (anti-grade) associated with tumor grade in our meta-analysis, CytoTRACE [70] score (as described in *ii*), and EMT mesenchymal genes: mesenchymal genes used to calculated epithelial to mesenchymal (EMT) scores in this and previous studies [71, 72]. *iv* Scatter plots highlighting correlations between LNM signatures and a selection of the tumor plasticity signatures shown in in the heatmap in *iii.* These correlations are shown within malignant cells (points) of the Puram scRNA-Seq dataset. Point colors correspond to the unsupervised cell clusters shown in the UMAP in *ii*, illustrating the expression of gene signatures within specific malignant cell subpopulations. Pearson correlation coefficients (R) and regression lines (dashed lines) indicate the correlation between expression scores. Asterisks indicate linear regression *p*-values for the association of expression scores: ***p < 0.001. **E** Deregulation of LNM-associated genes associated with epithelial dysplasia in oral premalignant lesions (OPLs). Box plots showing mean expression of anti-LNM genes and pro-LNM gene cluster L4 in OPLs (*n* = 86), using a publicly available dataset [78]. OPLs are stratified based on their stage of premalignant disease, with deeper color indicating higher risk lesions with advanced dysplasia

Pro-LNM genes were enriched for mitosis and cell cycle genes, particularly ones regulated by E2F transcription factors (TFs) and that are involved in the G2/M checkpoint (*CDK1, CCNB2, CHEK2*, *AURKA/B*) (Fig. 1B). They included proliferation markers *MKI67* and minichromosomal maintenance complex genes (*MCM3*, *MCM6-8*), as well as genes involved in DNA replication, recombination, and repair (*POLA1*, *RAD51*, *MSH6*). Strikingly, anti-LNM genes were enriched for squamous epithelial terminal differentiation processes, including cornification and keratinization. They included *IVL*, multiple keratins (*KRT2*, *KRT6B*, *KRT10*, *KRT16*, *KRT23*, *KRT75*, *KRT78*, *KRT80*), and nine contiguous kallikrein related peptidases (*KLK6*-*14*) within the 19q13 gene cluster, which regulate skin desquamification [83]. This suggests that LNM is strongly associated with loss of dedifferentiation or loss of epithelial identity within primary tumors. Indeed, anti-LNM and pro-LNM signatures were respectively strongly positively and negatively associated with tumor grade (i.e., level of pathological differentiation within malignant cells) across studies (Fig. 2A). Expression of LNM-associated genes displayed a stepwise progression from histologically normal tumor-adjacent tissue to tumors with increasing grades of dedifferentiation (Fig. 2B). We also identified genes associated with grade across studies (Additional file 1: Figure S5 and Additional file 2: Table S5). Three hundred twenty-four of 420 (77%) pro-LNM and 374 of 457 (82%) anti-LNM genes were also associated with grade (Additional file 2: Table S5), and gene meta-*z*’s for these associations were highly correlated (Pearson correlation coefficient = 0.63) (Fig. 2C).

Phenograph was used to cluster LNM-associated genes based on their co-expression within a combined dataset of 1642 primary HNCs (20 studies), which again identified six LNM-associated gene clusters. Three primarily consisted of anti-LNM genes (L1–L3), and three of pro-LNM genes (L4–L6) (Additional file 1: Figure S2B). All six clusters were strongly associated with tumor grade (Fig. 2A). Both anti-LNM clusters L1 and L2, the two major anti-LNM clusters that comprise 81% of anti-LNM genes combined, were overrepresented for epithelial differentiation genes (Additional file 2: Table S6). Thirty (14%) of the gene within cluster L2 overlapped with survival cluster S6 (Additional file 1: Figure S6; hypergeometric test *P* = 5 × 10^−31^), identifying a set of differentiation-related genes that are positively associated with survival and negatively associated with metastasis. Anti-LNM cluster L3 (*N* = 15 genes) was enriched for intracellular protein transport and RNA splicing.

Pro-LNM L5 genes were overrepresented for members of the oncogenic KRAS and IL2-STAT5 signaling pathways, as well as genes that regulate diverse immune processes including activation and proliferation of T cell and B lymphocytes, mononuclear cell differentiation, and immunoglobulin production. Pro-LNM cluster L6, the smallest LNM gene cluster (*N* = 14 genes) was (like anti-LNM cluster L3) overrepresented for RNA splicing, further suggesting a role of splicing regulation in metastasis.

### Disruption of the P53-DREAM pathway is a pro-LNM factor in HNC

**Fig. 3:**
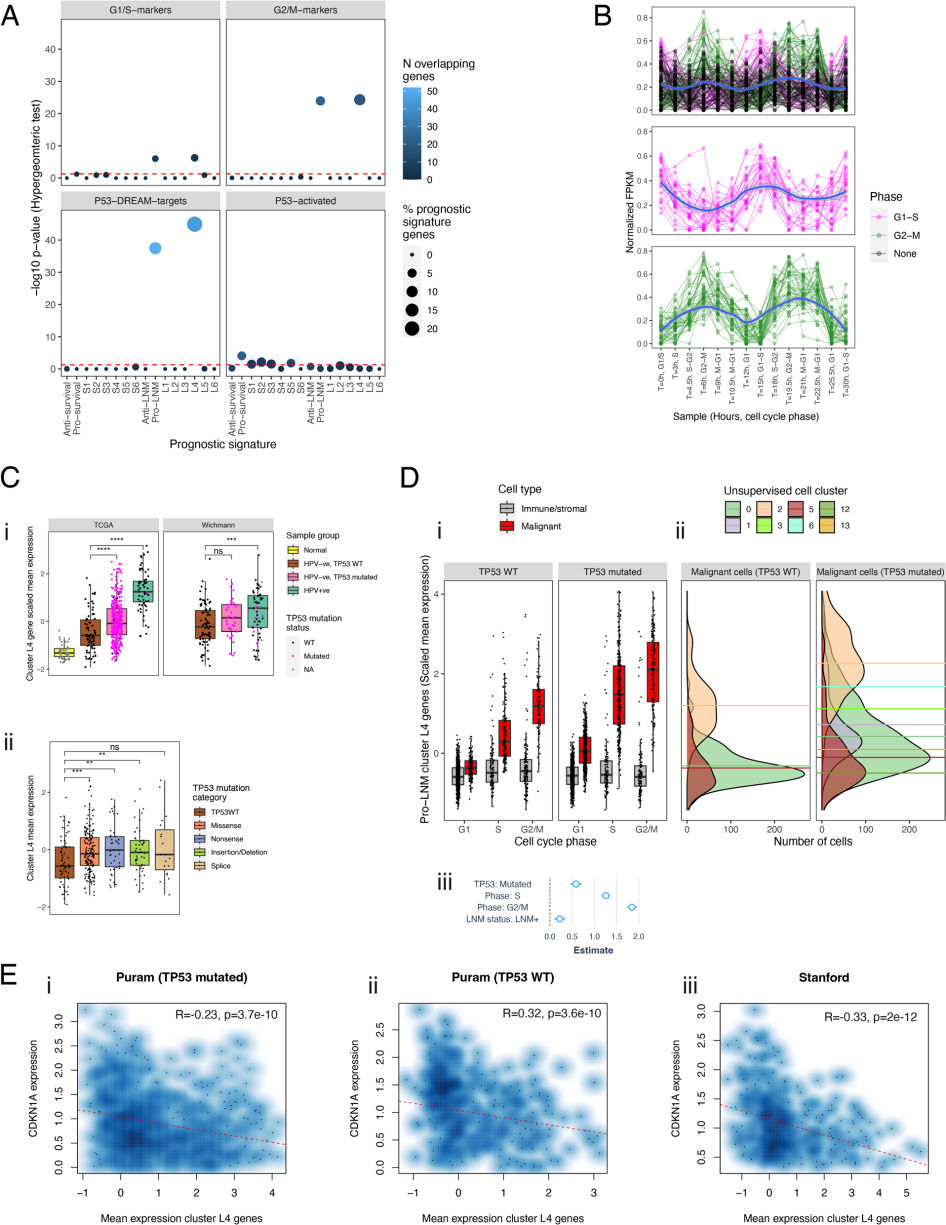
Deregulation of pro-LNM cluster L4 genes associated with p53-DREAM pathway-mediated repression: **A** Dot plot illustrating the overlap of prognostic gene signatures (survival and LNM-associated genes) with gene sets related to cell cycle and p53. Cell cycle gene sets include genes reported to be periodically expressed during G1 or S phase (G1/S markers, *n* = 15 genes) and G2 or M phase (G2/M-markers, *n* = 48 genes) across four cell lines [62]. P53-regulated gene sets include genes that are transcriptionally repressed by the p53-DREAM pathway (p53-DREAM targets, *n* = 202 genes) [60] and genes that are transcriptionally activated by p53 (P53-activated, *n* = 343 genes) [89]. Y-axes show − log10 *p*-values (hypergeometric tests) indicating the significance of overlaps between the prognostic gene signatures and the cell cycle/p53-related gene sets. Horizontal dashed red lines indicate significance thresholds (equivalent to *p* = 0.05). Point colors indicate the number of overlapping genes. Point sizes indicate the percentage of genes within the prognostic signature that overlap with the cell cycle/p53-related gene set. **B** Spaghetti plots showing expression changes of pro-LNM cluster L4 genes in Hela cells that were serially collected at 14 timepoints over the course of two cell cycles following cell synchronization (data derived from Dominguez et al. [62]). *Y*-axes represent expression (normalized FPKM) of genes (points). Lines connect each gene across timepoints. *X*-axis labels indicate the timepoint (number of hours since cell synchronization) as well as the phase at which cells were harvested. The top panel shows all genes within cluster L4, with point and line colors indicating the phase within which each gene was specifically expressed as reported by Dominguez et al. [62]*.* Genes labeled “None” represent non-periodic genes, i.e., genes that were stably expressed across phases. The middle and bottom panels show cluster L4 genes that were periodically expressed in G1/S and G2/M phases, respectively. These represent the genes shown in **A** (top panels). **C** Differential expression of cluster L4 genes between p53 inactivated and p53 proficient primary HNCs based on bulk gene expression analysis. *i* The box plots show mean expression (normalized counts) of cluster L4 genes in two HNC bulk gene expression studies including the *i* TCGA [16] and *ii* Wichmann [91] studies. Primary HNCs are stratified into groups based on their *TP53* mutation and human papillomavirus (HPV) status to illustrate differences of cluster L4 gene expression between p53 proficient and deficient HNCs. P53-proficient HNCs represent those that are HPV − ve and *TP53*^wt^, while p53-inactivated HNCs represent those with *TP53* mutations (TP53mut) or HPV positivity (HPV + ve). Expression of cluster L4 genes is also shown for tumor-adjacent normal tissue in the TCGA dataset. *ii* Expression of cluster L4 genes in HPV − ve primary HNCs of the TCGA study, comparing levels in *TP53*^wt^ HNCs with those in HNCs with *TP53* mutations of four major functional categories. TP53^mut^ HNCs were restricted to those with *TP53* mutations of only one functional category, to exclude ambiguity in cases with multiple mutations of different categories. Asterisks indicate Wilcoxon rank sum test *p*-values: *p < 0.05, **p < 0.01, ***p < 0.001. **D** Upregulation of cluster L4 genes in proliferating malignant cells of *TP53*^mut^ HNCs. *i* Box plots showing mean expression (normalized counts) of cluster L4 genes in cells (points) of *TP53*^wt^ and *TP53*^mut^ HNCs, with cells stratified by cell type (malignant or non-malignant) as well as cell cycle phase. *ii* Density plots of malignant cells shown in *i*, illustrating the distribution of cluster L4 gene expression within each unsupervised cell cluster. Cells are stratified into those derived from *TP53*^mut^ and *TP53*^wt^ HNCs. Horizontal dashed lines indicate mean cluster L4 gene expression within each cell cluster. *iii* Forest plot of linear regression coefficients (estimates) that indicate the association of cluster L4 gene expression with *TP53* mutation status and cell cycle phase within malignant cells (based on analysis of data shown in *i*). Coefficients were derived from a multiple linear regression model estimating the association of mean expression of cluster L4 genes (dependent variable) with two independent variables including *TP53* mutation status (the mutation status of the overall tumor) and cell cycle phase. Blue points and lines indicate coefficients and 95% confidence intervals, respectively. **E** Smoothed scatter plots illustrating the correlation of pro-LNM cluster L4 genes with *CDKN1A* within malignant cells. Scaled mean expression (normalized counts) of cluster L4 genes (*X*-axes) is plotted against *CDKN1A* expression (*Y*-axes) in malignant cells (points) of primary HNCs, including *i TP53*^mut^ HNCs of the Puram dataset, *ii TP53*^wt^ HNCs of the Puram dataset, and *iii* all HNCs of the Stanford dataset (TP53 mutation status unknown). Pearson correlation coefficients (R), linear regression *p*-values (P), and regression lines (red dashed lines) indicate associations between cluster L4 genes expression and *CDKN1A*

Pro-LNM gene cluster L4 was the largest pro-LM gene cluster and included six out of ten genes with the strongest pro-LNM associations. This cluster consisted of cell cycle and proliferation-related genes such as E2F TF target genes, which accounted for the enrichment of cell cycle genes in pro-LNM genes overall. Many of the genes within cluster L4 represent periodically expressed (oscillating) G2/M and G1/S phase genes [62] (Fig. 3A, B, Additional file 2: Table S6) indicating roles that are specific to different stages of the cell cycle. A subset of cell cycle genes that are repressed by E2F4/5 are indirectly regulated by p53 through the p53-p21-DREAM-CDE/CHR pathway [60, 85] (the “p53-DREAM” pathway). Since p53 inactivation occurs in most HNCs and is associated with LNM [1, 13], we investigated overlap of LNM signatures with a set of 201 p53-DREAM-repressed genes [60] (Fig. 3A). P53-DREAM targets were strikingly overrepresented within pro-LNM cluster L4 (Fig. 3A) and displayed a much stronger enrichment within pro-LNM genes than any of the MsigDB gene sets previously analyzed (normalized GSEA score = 3.5, FDR-adjusted *p*-value = 4 × 10^-52^). We tested the hypothesis that cluster L4 genes are overexpressed in HNCs due to p53 inactivation by analyzing their expression in relation to both *TP53* mutations and HPV status (Fig. 3C), since p53 is targeted for ubiquitination-mediated degradation by the HPV E6 oncoprotein [86]. Indeed, cluster L4 genes were upregulated in both HPV + ve tumors and TP53^mut^/HPV − ve HNCs relative to TP53^wt^/HPV − ve HNCs, indicating that these genes might be upregulated in HNC due to loss of repression by p53-DREAM. Moreover, cluster L4 genes were upregulated in HNCs with different functional categories of *TP53* mutation (Fig. 3C). This is consistent with the hypothesis that they are upregulated due to loss of p53 TF activity, which is associated with all *TP53* mutation types, rather than acquired functions of mutant p53, which are conferred by specific (usually missense) *TP53* mutations [87]. Importantly, p53 is primarily a transcriptional activator and represses transcription indirectly by transcriptionally activating *CDKN1A* (encoding p21), whose cyclin-dependent kinase-inhibitory activity facilitates assembly of the DREAM complex [60, 88]. Indeed, *CDKN1A* was negatively associated with LNM in our meta-analysis (included in anti-LNM cluster L1), suggesting that p21 could prevent metastasis by mediating transcriptional repression of P53-DREAM target genes within cluster L4. Importantly, other than *CDKN1A*, LNM-associated genes were not significantly enriched for genes that are directly activated by p53 [89], even though pro-survival genes significantly overlapped with p53-activated genes (*P* = 8 × 10^−5^) (Fig. 3A). Indeed, enrichment of pro-survival genes for p53-activated genes (e.g., *TP53INP1*, *TP73*, *BTG3*) could be expected, since *TP53* mutations are associated with adverse survival [16]. Together these observations suggest that p53-inactivation supports LNM by upregulating genes that are repressed by p53-DREAM, while downregulation of p53-activated genes influences survival thorough LNM-independent mechanisms, such as by conferring therapy resistance [90].

Interestingly, cluster L4 genes were expressed at particularly high levels in HPV + ve HNCs (Fig. 3C), which could be explained due to inactivation of both p53 and DREAM by the HPV E6 [86, 92]and E7 [93] oncoproteins, as well as HPV E7-mediated repression of the tumor suppressor Rb1 [94], which transcriptionally represses a subset of proliferation genes that are also repressed by DREAM. Upregulation of these pro-LNM genes could explain the paradoxical observation that HPV + ve HNCs have particularly high rates of LNM, despite their favorable prognosis [95, 96]. Moreover, high expression of cluster L4 genes in HPV + ve HNCs could account for why these genes were not associated with survival, since they are associated with both adverse (LNM and p53 inactivation) and favorable (HPV positivity) prognostic factors. Indeed, while not associated with survival in HNC specifically, they were strongly adversely associated with survival in prior pan-cancer analysis [79] (Additional file 1: Figure S3B).

### Specific cell types in the HNC TME express survival-associated genes

Identification of cell types that express prognostic genes could yield insight into their roles in disease progression. We sought to identify cells types that express survival-associated genes using two primary HNC scRNA-Seq datasets: a set of five HPV − ve primary HNCs that we profiled on the 10X Genomics platform (Stanford scRNA-Seq dataset [63]); and a published scRNA-Seq dataset of nine HPV − ve primary HNCs profiled using Smart-Seq2 technology (Puram dataset [67]) (Additional file 1: Figure S7 & S8). In each scRNA-Seq dataset, we identified the cell types that most highly expressed each prognostic gene (Table 3, Additional file 1: Figure S9, Additional file 2: Table S4). We further validated our observations of cell type-specific expression by analyzing the expression of survival and LNM genes in bulk RNA-Seq data of four cell populations that we flow sorted from primary HNCs (Table 3, Additional file 1: Figure S10, Additional file 2: Table S4) (Stanford bulk RNA-Seq dataset). These included malignant cells (*n* = 13), fibroblasts (*n* = 10), lymphocytes (*n* = 15), and endothelial cells (*n* = 12). To confirm the enrichment of target cell types by flow cytometry, CIBERSORTx was applied to infer the fractions of cell types within the Stanford bulk RNA-Seq dataset (Additional file 1: Figure S10B).

**Table 3:**
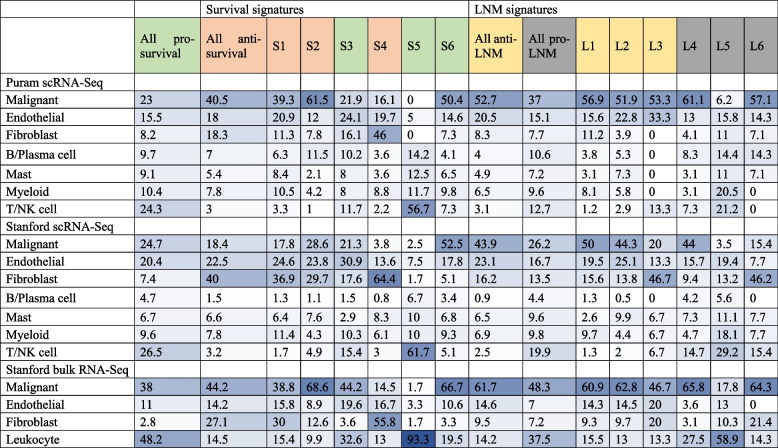
Percentages of genes in each prognostic signature that were highest expressed in each cell type in scRNA-Seq and flow-sorted cell datasets

Expression of anti-survival cluster S1 and S2 genes were expressed in both malignant and mesenchyme-derived stromal cells in all three datasets (Table 3, Additional file 1: Figure S9 & S10). Consistent with their enrichment for EMT drivers (e.g., *SNAI2* and *ITGB6)*, expression of cluster S1 genes was correlated (Pearson *r* = 0.55, *P* < 2.2 × 10^-16^) with “EMT score,” a commonly used measure of EMT based on the expression of epithelial and mesenchymal genes [72] (Figure S11). Within malignant cells, expression of cluster S1 genes was strongly elevated within a distinct subpopulation of cells that displayed high EMT score, consistent with the existence of an aggressive mesenchymal malignant cell population within HNC. In contrast with cluster S1 genes, cluster S4 genes were largely restricted to fibroblasts. They included known fibroblast markers (e.g., *FAP*, *FN1*, *SERPINH1*) [66] and were mostly expressed in a subset termed cancer-associated fibroblast 1 (CAF1) [67] (Additional file 1: Figure S12). Indeed, recent single-cell studies have indicated that many adversely prognostic genes that were previously considered to represent cancer EMT markers are highly expressed in cancer-associated fibroblasts [97].

Expression of pro-survival cluster S3 genes were ubiquitously expressed across cell types, while S5 genes were restricted to cytotoxic T cells and other lymphocytes (Table 3, Additional file 1: Figure S9 & S10). Consistent with enrichment for epithelial markers, pro-survival cluster S6 was restricted to a minor subpopulation of non-proliferating (G1 phase) malignant cells that was particularly well differentiated, as indicated by high expression of anti-grade genes as well as a reported epithelial differentiation signature [67] (Table 3, Additional file 1: Figures S9, S10, & S13).

### LNM genes are associated with malignant cell dedifferentiation and proliferation linked to loss of P53-DREAM-mediated repression

**Fig. 4:**
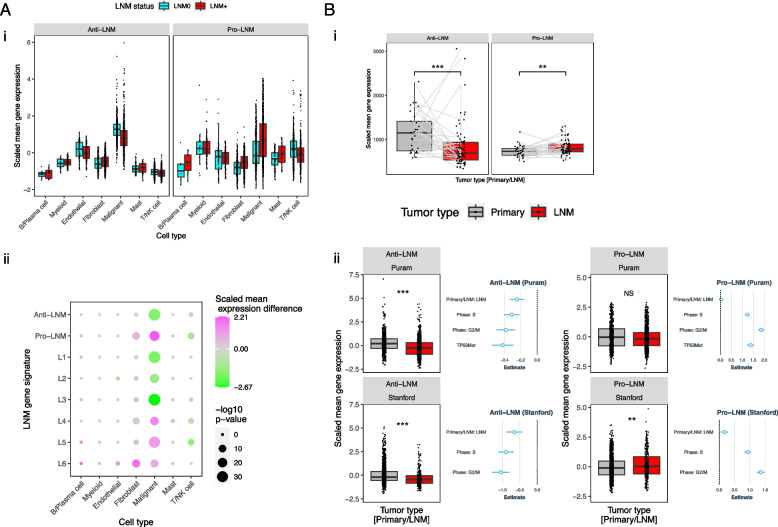
Deregulation of LNM gene signatures in lymph node metastasis (LNM) positive primary HNCs and lymph node metastases. Differential expression of LNM gene signatures in **A** lymph node-positive primary HNC (LNM +) relative to LNM- primary HNC (LNM0) and **B** lymph node metastases (i.e., metastatic tumors) relative to patient-matched primary HNCs. The LNM gene signatures shown consist of genes negatively (anti-LNM) and positively (pro-LNM) associated with LNM status in our meta-analysis, as well as genes with six unsupervised LNM gene clusters (L1-6). Expression scores were calculated for each LNM gene signature (i.e., set of LNM-associated genes) as the mean of expression (normalized counts) of all genes within the signature. **A** Deregulation of LNM gene signatures in LNM + primary HNCs. *i* Box plots of LNM gene signature scores in LNM0 (Cyan, *N* = 3) and LNM + (Red, *N* = 6) primary HNCs, within the Puram scRNA-Seq dataset, with cells (points) stratified by LNM status and cell type. *ii* Heatmap of scaled mean differences between LNM + and LNM0 primary HNCs of LNM gene signature scores, within each cell type, in the Puram scRNA-Seq dataset. The point color gradient indicates the scaled mean differences of gene signature scores between LNM + and LNM0 tumors. Point sizes indicates negative log10 *p*-values (Wilcoxon rank sum test). **B** Box plots of LNM-associated gene signatures in primary HNCs and patient-matched LNMs in *i* bulk and *ii* single-cell RNA-Seq datasets. *i* Box plots of LNM gene signature scores in primary HNCs (gray, *N* = 29) and patient-matched LNMs (red, *N* = 72) of the Huang bulk RNA-Seq dataset (EGAD00001004489) [98]. Points represent patient samples. LNM gene signature scores are calculated as mean expression (normalized counts) of genes within each LNM-associated gene signature (i.e., set of genes). Asterisks indicate Wilcoxon rank sum test *p*-values: **p < 0.01, ***p < 0.001. *ii* Box plots of LNM gene signature scores in malignant cells (points) of primary HNCs (gray) and patient-matched LNMs (red) in two scRNA-Seq study datasets, including the Puram and Stanford datasets. Cells are stratified by tumor type (primary tumor or LNM), with groups consisting of malignant cells of combined patient samples of each tumor type. Asterisks indicate linear regression *p*-values for association of the LNM gene signatures with tumor type, adjusted for covariates known to correlate with these genes. These include cell cycle phase and *TP53* somatic mutation status (In the Puram dataset for which *TP53* mutation data was available). ***p < 0.001, **p < 0.01. Forest plots to the right of each box plot indicate regression coefficients (estimates) derived from the multiple linear regression models. These models estimate associations of LNM gene signature scores (dependent variable) with sample type (independent variable) adjusted for cell cycle phase (covariate) and TP53 mutation status (covariate)

Analysis of LNM gene signatures in scRNA-Seq data confirmed downregulation of anti-LNM genes and upregulation of pro-LNM genes in combined cells of LNM + primary HNCs (*N* = 6) relative to those of LNM0 primary HNCs (*N* = 3), adjusting for cell type, cell cycle phase, and TP53 mutation status (Fig. 4A). This validated the association of the meta-analysis-derived genes with LNM status in combined cell types, analogous to bulk gene expression data. Interestingly, however, analyses within each cell type revealed strong downregulation of all three anti-LNM gene clusters, and upregulation of all three pro-LNM clusters, within malignant cells of LNM + HNCs; however, no consistent pattern of deregulation was observed in any other cell type (Fig. 4A). This suggests that the LNM-associated genes are deregulated primarily (or specifically) within malignant cells of LNM + HNCs. Consistent with this, LNM-associated genes, both anti-LNM and pro-LNM genes, were primarily expressed within malignant cells (Figs. 2D and 4A, Additional file 1: Figures S9B, S10, & S13), in contrast with the heterogenous expression of survival-associated genes across cell types. Both major clusters of anti-LNM genes (L1 and L2) were restricted to the well-differentiated malignant cell cluster that also expressed pro-survival cluster S6 genes (with which cluster L2 overlapped) (Table 3, Fig. 2D, Additional file 1: Figures S9B, S10, & S13). This concurs with the epithelial differentiation-related functions of these genes. Anti-LNM cluster L3 genes were ubiquitous across cell types, consistent with their roles in basic cellular processes.

Pro-LNM cluster L4, the largest pro-LNM gene cluster, was primarily expressed in malignant cells (Table 3, Additional file 1: Figures S9, S10), particularly those in G2/M cell cycle phase (Figs. 2D and 3D, & Additional file 1: S13), consistent with our earlier findings. Moreover, they were upregulated in malignant cells of *TP53* mutated (*TP53*^mut^) HPV − ve HNCs (*N* = 5) relative to TP53 wild-type (*TP53*^wt^) HPV − ve HNCs (*N* = 3) (Fig. 3D), implying that their overexpression is caused by p53 inactivation. Furthermore, within malignant cells, cluster L4 genes were negatively correlated with *CDKN1A* (Fig. 3E), consistent with their being transcriptionally repressed by the p53-DREAM pathway [88]. Interestingly, they were anticorrelated with *CDKN1A* expression within malignant cells of both *TP53*^mut^ and *TP53*^wt^ HNCs, suggesting that in the absence of *TP53* mutations or HPV, downregulation of p21 by other mechanisms such as TP53 deletion [99] could disrupt DREAM-mediated repression of pro-LNM genes.

Pro-LNM Cluster L5 genes were ubiquitously expressed, consistent with their diverse functional repertoire (Table 3, Additional file 1: Figures S9 & S10). Many cluster L5 genes were primarily expressed in leukocytes, confirming a previous report of an immune gene signature that was associated with LNM [19]. Cluster L6 genes were ubiquitously expressed across cell types (Table 3, Additional file 1: Figure S9 & S10), consistent with their roles in RNA splicing.

Taken together, our findings indicate that LNM-associated genes are primarily deregulated within the malignant cells. This is consistent with the functional roles of LNM-associated genes in epithelial differentiation and cell cycle regulation, as well as the observation that they are associated with tumor grade, a measure of dedifferentiation of malignant cells.

### Less differentiated LNM-associated malignant cells express non-EMT-related stemness genes

Interestingly, in both scRNA-Seq datasets, the subset of L4-expressing malignant cells that lacked expression of differentiation markers also highly expressed “stemness signatures,” including genes positively associated with tumor grade in HNC and embryonic stem cell (ESC)-specific genes (Fig. 2D, Additional file 1: Figure S13). This malignant cell subpopulation displayed high transcriptional diversity—a hallmark of pluripotent cells—as assessed by CytoTRACE analysis [70] (Fig. 2D, Additional file 1: Figure S13). Within malignant cells, cluster L4 genes were linearly correlated with dedifferentiation and stemness signatures (Fig. 2D). Taken together, these findings suggest that a subset of malignant cells expressing LNM cluster L4 genes could be cancer stem-like cells that have the potential to seed metastases [100].

Pro-LNM cluster L4 genes and anti-LNM genes displayed a “mutually exclusive” expression pattern in scRNA-Seq (Additional file 1: Figure S14), suggesting an antagonistic relationship between them. This inverse association is consistent with the general observation that upregulation of cell cycle genes is inversely correlated with expression of cell type-specific differentiation-related genes across multicellular organisms [101, 102]. Thus, loss of epithelial differentiation transcriptional programs in malignant cells might represent an oncogenic switch to a proliferative state occurring as a secondary consequence of loss of p53-DREAM-mediated repression. Importantly, while EMT has been implicated in both LNM and stemness [103], in our analysis pro-LNM genes did not include EMT or mesenchyme-related genes, and did not correlate with EMT score (Fig. 2D & Additional file 1: S11B).

### Enhanced deregulation of LNM-associated genes after lymph node metastasis

Our LNM meta-analysis identified genes that are deregulated in primary tumors of LNM + HNC cases; we next investigated whether they are further deregulated after metastasis, by comparing their expression in LNMs (metastatic tumors) relative to patient-matched primary tumors. We first investigated this at the patient population level by comparing mean expression of pro-LNM genes and anti-LNM between bulk RNA-Seq profiles of primary HNCs (*N* = 29), and patient-matched LNMs (*N* = 72), using a dataset published by Huang et al. [98]. This revealed strong downregulation of anti-LNM genes in LNMs relative to primary tumors, coupled with modest but statistically significant upregulation of Pro-LNM genes (Fig. 4B). Since our scRNA-Seq analyses indicated that LNM-associated genes are deregulated primarily within malignant cells, we next compared their expression between malignant cells of primary HNCs and patient-matched LNMs within the Puram and Stanford scRNA-Seq datasets. This confirmed downregulation of anti-LNM genes in malignant cells of LNMs relative to those of primary tumors in both datasets, adjusted for known modifiers of LNM gene expression including cell cycle phase and *TP53* mutation status (In the Puram dataset where *TP53* mutation status was available) (Fig. 4B). Pro-LNM genes were marginally upregulated in malignant cells of LNMs within the Stanford dataset, but not the Puram dataset; therefore we could not confirm the upregulation of pro-LNM genes in LNMs observed in bulk RNA-Seq data.

We investigated the expression patterns of LNM-associated genes within LNMs by analyzing their expression in combined LNMs and primary HNCs of the Puram and Stanford scRNA-Seq datasets. Consistent with our observations in primary HNCs, pro-LNM gene expression was highest in a stem-like, proliferating malignant cell cluster that consisted of both primary tumor and LNM-derived malignant cells (Additional file 1: Figure S14). This indicates that LNMs maintain a subpopulation of stem-like malignant cells after metastasis. Also consistent with primary HNCs, anti-LNM genes were highest expressed within a well-differentiated malignant cell cluster within LNMs; however, their expression was lower within malignant cells of each unsupervised cell cluster in LNMs compared to those of the same cluster in primary HNCs (Additional file 1: Figure S14). This indicates that anti-LNM genes are generally downregulated throughout the malignant cell compartments of LNMs.

### Deregulation of LNM-associated genes as an early tumorigenic event

Our observation that pro-LNM genes are upregulated in p53-inactivated malignant cells led us to hypothesize that transcriptional repression these p53-DREAM target genes could be lost during early tumorigeneses, since p53 inactivation occurs in premalignant lesions and is understood to cause tumorigenesis in HNC [17, 104, 105]. To investigate this, we analyzed expression of LNM-associated genes in 86 oral premalignant lesions (OPLs) [78]. Indeed, we found that pro-LNM cluster L4 genes were strongly upregulated with advancing stages of premalignant disease progression and increasing epithelial dysplasia, a histological phenotype used to grade OPLs that identifies OPLs at higher risk of malignant transformation [106] (Fig. 2E). Conversely, anti-LNM were negatively associated with progression (Fig. 2E). Together these findings indicate that upregulation of cluster L4 p53-DREAM target genes is concomitant with epithelial dedifferentiation, precedes malignant transformation, and could be an early driver of LNM.

## Discussion

Robust identification of pathways and cell types with clinical prognosis in HNC can yield insights into the biology of HNC progression and be used to nominate targeted therapies. Through large-scale meta-analysis, we identified genes associated with survival and LNM. Unsupervised clustering applied to these genes highlighted clusters of co-expressed genes which were associated with distinct pathways. Analysis of these prognostic gene clusters in HNC scRNA-Seq and flow-sorted cells indicated that some were associated with distinct cell subtypes, revealing cell subtypes and processes that influence clinical outcomes.

A key finding is that genes associated with LNM status are primarily deregulated within the malignant cells and that their deregulation is intrinsically tied to epithelial dedifferentiation, as indicated by their associations with grade and stemness within malignant cells. Genes negatively associated with LNM (anti-LNM genes) were enriched for epithelial-specific functions and are expressed within well-differentiated malignant cells. Conversely, pro-LNM genes were strongly associated with grade; and the largest cluster of them (cluster L4) was primarily expressed in undifferentiated malignant cells. Dedifferentiation is a hallmark of cancer that is indicative of aggressiveness and poor prognosis across cancer types [107, 108]. This subset of undifferentiated malignant cells expressing pro-LNM genes is thus consistent with an aggressive subpopulation of metastatic cancer stem-like cells [100]. Our findings postulate that epithelial dedifferentiation is a major driver of LNM in HNC and could represent an early driver, since LNM-associated genes were associated with epithelial dysplasia in OPLs.

We identified loss of p53-DREAM-mediated repression as a potential initiating mechanism of LNM in HNC, since pro-LNM cluster L4 genes were overrepresented for known targets of this pathway and were overexpressed in malignant cells of p53^mut^ HNCs, as well as in HPV + ve HNCs, where it is disrupted by HPV [86, 93]. Furthermore, expression of this gene cluster was negatively associated with expression of *CDKN1A*, an essential mediator of p53-DREAM-mediated repression, which was itself negatively associated with LNM. Since *TP53* mutations and HPV infection represent tumor initiating events in HNC [4, 109], p53 inactivation likely represent the initial cause of deregulation of cluster L4 genes, particularly since they were overexpressed in high-grade OPLs, in which *TP53* mutations frequently occur and are understood to predispose to malignant transformation [17, 104]. The hypothesis that p53 inactivation leads to LNM is supported by experimental evidence in animal models [110, 111], as well as by the observations that *TP53* mutations are associated with LNM [19, 20] and extranodal extension [112]. While p53 regulates many oncogenic processes both through its TF activity as well as transcription-independent mechanisms [113–115], *TP53* mutations are understood to promote cancer primarily by disrupting TF activity, since virtually all disrupt DNA binding [87, 116]. Our findings suggest that loss of p53 TF activity drives LNM specifically due to loss of p53-DREAM-mediated repression, since genes that are activated by p53, apart from *CDKN1A*, were not generally associated with LNM, even though they were associated with longer survival.

**Fig. 5:**
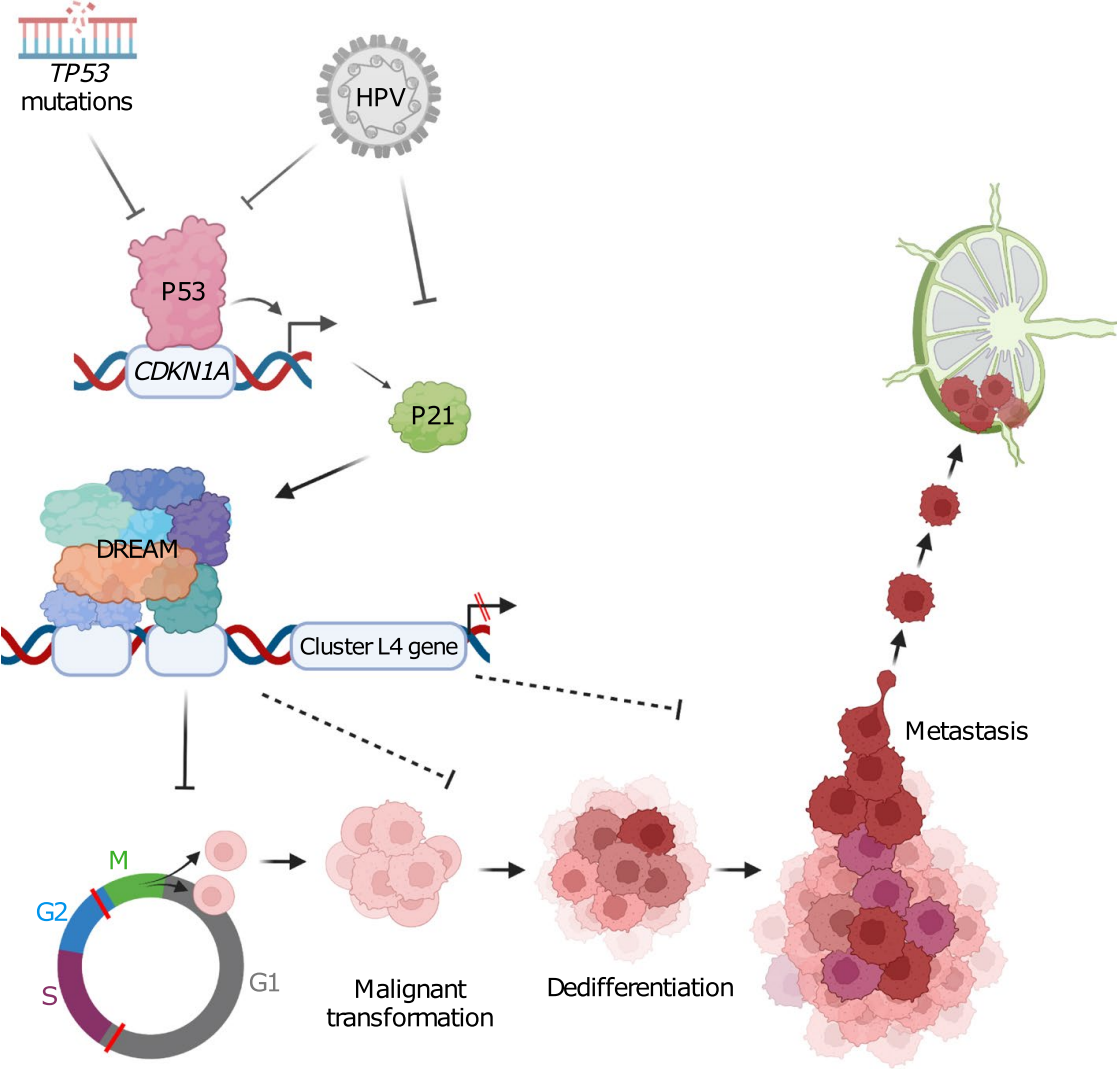
Proposed model of the primary cause of LNM in HNC: In normal squamous epithelial cells, p53 induces cell cycle arrest by indirectly repressing cell cycle genes, via the p53-DREAM pathway [85]. Specifically, the p53-DREAM pathway represses transcription of G1/S and G2/M phase cell cycle genes [60], such as those within cluster L4, the most significant subset of pro-LNM genes that was identified in this study. Abrogation of the p53-DREAM pathway due to either *TP53* mutations or HPV [93] causes overexpression of cluster L4 genes as an early tumorigenic event (prior to malignant transformation), resulting in cellular proliferation. Upregulation of cluster L4 genes appears to cause epithelial differentiation, which is associated with epithelial dysplasia in premalignant lesions, dedifferentiation in HNC populations, and stemness within malignant cells. Dedifferentiation could be caused by upregulation of cell cycle genes, resulting in a switch from a differentiated to a proliferative state [101, 102]. Dedifferentiation could be also be induced by overexpression of genes in cluster L4 that encode stemness-related epigenetic modifying enzymes such as *DNMT1* [118] and *SUZ12* [119], or other stemness drivers such as *BIRC5* [120] and *RFC4* [121]. Our findings suggest that dedifferentiation promotes LNM by giving rise to cancer stem-like cells that have increased potential to seed metastasis [122]

Anti-LNM genes could be downregulated as a secondary consequence of loss of p53-DREAM-mediated repression, as their expression is lost in the stem-like malignant cells that express pro-LNM genes. Moreover, the pro-LNM proliferation-related genes were also found to be strongly associated with tumor grade, indicating that proliferation coincides with stable epithelial dedifferentiation. This pattern of mutual exclusion is consistent with the observation across multicellular organisms that expression of genes that promote cell cycle progression and proliferation is inversely associated with expression of cellular differentiation-related genes [101, 102]. While the mechanisms underlying this phenomenon are not fully understood, terminal differentiation is thought to require exit from the cell cycle, which is facilitated by repression of cell cycle genes by DREAM and its E2F components [102, 117]. Our findings therefore lead us to postulate a model wherein loss of p53-DREAM-mediated repression of cell cycle arrest causes hyperproliferation of malignant cells, in turn causing epithelial dedifferentiation and stemness by antagonizing expression of epithelial pathways (Fig. 5).

Genes downregulated in LNM + primary tumors (anti-LNM genes) were further downregulated in malignant cells of lymph node metastases. This suggests that loss of these epithelial differentiation-related genes not only predisposes to metastasis but could contribute to later stages of metastasis. Moreover, this indicates that dedifferentiation is enhanced during metastasis in HNC, in contrast with the hypothesis that pro-metastatic cellular plasticity is reversible after metastatic colonization [123–125]. LNMs were found to contain a subpopulation of stem-like malignant cells transcriptionally similar to those observed in primary tumors, albeit with lower expression of anti-LNM genes; these cells therefore feature particularly metastatic transcriptomes, suggesting their potential to seed further metastases.

Favorably survival-associated genes included a subset of the epithelial differentiation-related genes that were negatively associated with LNM; genes that presumably prolong survival by preventing metastasis. Despite this, most survival-associated genes were involved in immune and stroma-related processes that were not associated with LNM. Indeed, over half of survival-associated genes were primarily expressed in non-malignant cells, indicative of the importance of tumor immune and stromal microenvironments in HNC progression [26, 80–82, 126]. Some of our findings were expected, such as a major role for T cells/NKT cell-expressed gene programs in promoting survival. More surprising was the striking overrepresentation of mesenchyme-related genes among anti-survival genes, including many that have been implicated in EMT. A subset of these genes, particularly those within anti-survival cluster S1, were highly expressed in malignant cells that also had high EMT score. But cluster S1 genes were also high in fibroblasts, which have increasingly been implicated in HNC progression [127, 128]. Additionally, anti-survival genes in cluster S4 were mostly restricted to fibroblasts, and therefore cannot be related to malignant cell EMT. These genes were overrepresented for ECM component genes, further suggesting that they promote HNC progression by modulating fibroblast remodeling of the ECM [129–131]. Alternatively, CAFs could promote HNC progression by expressing growth factors that promote proliferation and growth of malignant cells, or by modulating the immune system to avoid detection of malignant cells [132]. While our findings support a possible role for EMT in HNC survival, they are inconsistent with the hypothesis that EMT promotes either LNM or stemness, despite the widespread hypothesis that EMT causes metastasis by giving rise to stem-like malignant cells [82, 103]. Indeed, a recent report showed that EMT scores were generally not associated with metastasis after controlling for stromal expression of mesenchymal genes [97].

## Conclusions

In conclusion, our results suggest that LNM is primarily driven by loss of p53-DREAM-mediated repression resulting in proliferation and EMT-independent dedifferentiation of malignant cells, while patient survival is influenced by epithelial differentiation in addition to tumor microenvironmental factors. Experimental studies are needed to confirm a causal pro-metastatic role of p53-DREAM target genes in HNC, which would nominate them as potential therapeutic targets for this deadly disease.

### Supplementary Information

**Additional file 1: Supplementary Methods & Results. Supplementary Discussion. Supplementary References.** **Figure S1. **FlowJo contour plots illustrating the gating strategy that was used to isolate four cell types from head and neck cancer tumors using fluorescence-activated cell sorting (FACS): Cells were analyzed using FlowJo V. 10.6.1 and first gated on single cell size using FSC width and height and cell granularity using SSC width and height. **Figure S2. **Unsupervised clustering of survival and LNM-associated genes based on co-expression. **Figure S3.** Pan-cancer survival meta-z scores of genes that were associated with survival and lymph node metastasis (LNM) in head and neck cancer (HNC). **Figure S4. **Independence of prognostic signatures from potential confounding factors. **Figure S5.** Meta-analysis-based identification of genes associated with tumor grade in HNC. **Figure S6.** Overlap of genes between LNM gene clusters and survival gene clusters. **Figure S7.** UMAP representations of primary HNCs within the Stanford scRNA-Seq dataset. **Figure S8.** UMAP representations of the Puram scRNA-Seq dataset. **Figure S9. **Expression of prognostic gene signatures in two primary HNC scRNA-Seq datasets. **Figure S10. **Expression of prognostic gene signatures in four major cell types, as indicated by bulk RNA-Seq-derived transcriptional profiles of flow sorted cells. **Figure S11.** Correlations of prognostic gene signatures with an epithelial to mesenchymal transition (EMT) transcriptional score within primary HNC malignant cells. **Figure S12. **UMAPs (Seurat feature plots) showing fibroblast and myofibroblast gene signatures in primary HNCs of the Puram and Stanford primary HNC scRNA-Seq datasets. **Figure S13.** Expression of lymph node metastasis (LNM) and differentiation-related gene signatures in two primary HNC single cell RNA-Seq datasets (Supplementary to figure 2D). **Figure S14. **Expression of LNM-associated gene signatures within distinct subpopulations of malignant cells in primary HNCs and patient-matched lymph node metastases (LNMs).

**Additional file 2: Table S1.** Details of all curated head and neck cancer gene expression studies. **Table S2.** HNC patients within the Stanford bulk RNA-Seq dataset (Bulk RNA-sequencing of flow sorted-cells). **Table S3.** Antibodies used for flow cytometry. **Table S4.** Survival and LNM-associated genes. **Table S5.** Genes associated with cancer grade based on a meta-analysis. **Table S6.** Top 10 gene sets that most significantly overlapped with each prognostic gene signature.

## Data Availability

The Stanford bulk RNA-Seq dataset is accessible from Gene Expression Omnibus (Accession number: GSE113839) [133]. The Stanford scRNA-Seq dataset, which was recently described [63], is also available from GEO (Accession number: GSE140042). All other datasets that were analyzed and are publicly available. Accession numbers for datasets that were analyzed as part of these meta-analyses are provided in Table 1 and Additional file 2: Table S1. The uniformly processed gene expression and accompanying clinical datasets used to perform meta-analyses are publicly available at Zenodo [134]: https://zenodo.org/record/7679088. *Publicly available datasets analyzed* Publicly available datasets analyzed within this study were accessed from *Gene Expression Omnibus* [135], *Array Express* [136], and the *European Genome Phenome Archive* [137]. These datasets are accessible using the following accession codes (with references and URL links): Gene Expression Omnibus: GSE113839 [133]—https://www.ncbi.nlm.nih.gov/geo/query/acc.cgi?acc=GSE113839 GSE140042 [63]—https://www.ncbi.nlm.nih.gov/geo/query/acc.cgi?acc=GSE140042 GSE103322 [67]—https://www.ncbi.nlm.nih.gov/geo/query/acc.cgi?acc=GSE103322 GSE26549 [78]—https://www.ncbi.nlm.nih.gov/geo/query/acc.cgi?acc=GSE26549 GSE39366 [37]—https://www.ncbi.nlm.nih.gov/geo/query/acc.cgi?acc=GSE39366 GSE2837 [46]—https://www.ncbi.nlm.nih.gov/geo/query/acc.cgi?acc=GSE2837 GSE31056 [48]—https://www.ncbi.nlm.nih.gov/geo/query/acc.cgi?acc=GSE31056 GSE65858 [19]—https://www.ncbi.nlm.nih.gov/geo/query/acc.cgi?acc=GSE65858 GSE41613 [39]—https://www.ncbi.nlm.nih.gov/geo/query/acc.cgi?acc=GSE41613 GSE42743 [39]—https://www.ncbi.nlm.nih.gov/geo/query/acc.cgi?acc=GSE42743 GSE33205 [51]—https://www.ncbi.nlm.nih.gov/geo/query/acc.cgi?acc=GSE33205 GSE41116 [42]—https://www.ncbi.nlm.nih.gov/geo/query/acc.cgi?acc=GSE41116 GSE85195 [43]—https://www.ncbi.nlm.nih.gov/geo/query/acc.cgi?acc=GSE85195 GSE3292 [44]—https://www.ncbi.nlm.nih.gov/geo/query/acc.cgi?acc=GSE3292 GSE95805 [45]—https://www.ncbi.nlm.nih.gov/geo/query/acc.cgi?acc=GSE95805 GSE23558 [47]—https://www.ncbi.nlm.nih.gov/geo/query/acc.cgi?acc=GSE23558 GSE2379 [49]—https://www.ncbi.nlm.nih.gov/geo/query/acc.cgi?acc=GSE2379 GSE75538 [138]—https://www.ncbi.nlm.nih.gov/geo/query/acc.cgi?acc=GSE75538 GSE75539 [138]—https://www.ncbi.nlm.nih.gov/geo/query/acc.cgi?acc=GSE75539 GSE27020 [38]—https://www.ncbi.nlm.nih.gov/geo/query/acc.cgi?acc=GSE27020 GSE30784 [139]—https://www.ncbi.nlm.nih.gov/geo/query/acc.cgi?acc=GSE30784 GSE23036 [50]—https://www.ncbi.nlm.nih.gov/geo/query/acc.cgi?acc=GSE23036 GSE6791 [140]—https://www.ncbi.nlm.nih.gov/geo/query/acc.cgi?acc=GSE6791 GSE10121 [52]—https://www.ncbi.nlm.nih.gov/geo/query/acc.cgi?acc=GSE10121 GSE78060 [53]—https://www.ncbi.nlm.nih.gov/geo/query/acc.cgi?acc=GSE78060 GSE9844 [54]—https://www.ncbi.nlm.nih.gov/geo/query/acc.cgi?acc=GSE9844 GSE2280 [55]—https://www.ncbi.nlm.nih.gov/geo/query/acc.cgi?acc=GSE2280 GSE6631 [56]—https://www.ncbi.nlm.nih.gov/geo/query/acc.cgi?acc=GSE6631 GSE3524 [57]—https://www.ncbi.nlm.nih.gov/geo/query/acc.cgi?acc=GSE3524 ArrayExpress E-MTAB-1328 [40]—https://www.ebi.ac.uk/biostudies/arrayexpress/studies/E-MTAB-1328 European Genome Phenome Archive EGAS00001003233 [98]—https://ega-archive.org/studies/EGAS00001003233 The Cancer Genome Atlas TCGA data [16] – TCGA data was accessed using TCGAbiolinks [31]. The code used to perform the meta-analyses, as well as the code used to process and analyze the bulk and single-cell RNA-Seq datasets are available at GitHub [141].
