# Supplementary material for: Loss of p53-DREAM-mediated repression of cell cycle genes as a driver of lymph node metastasis in head and neck cancer
Source: Genome Med. 2023 Nov 17;15:98. doi: 10.1186/s13073-023-01236-w (PMC10656821; doi:10.1186/s13073-023-01236-w)
Supplement: Supplementary file 1 — Additional file 1: Supplementary Methods & Results. Supplementary Discussion. Supplementary References. Figure S1. FlowJo contour plots illustrating the gating strategy that was used to isolate four cell types from head and neck cancer tumors using fluorescence-activated cell sorting (FACS): Cells were analyzed using FlowJo V. 10.6.1 and first gated on single cell size using FSC width and height and cell granularity using SSC width and height. Figure S2. Unsupervised clustering of survival and LNM-associated genes based on co-expression. Figure S3. Pan-cancer survival meta-z scores of genes that were associated with survival and lymph node metastasis (LNM) in head and neck cancer (HNC). Figure S4. Independence of prognostic signatures from potential confounding factors. Figure S5. Meta-analysis-based identification of genes associated with tumor grade in HNC. Figure S6. Overlap of genes between LNM gene clusters and survival gene clusters. Figure S7. UMAP representations of primary HNCs within the Stanford scRNA-Seq dataset. Figure S8. UMAP representations of the Puram scRNA-Seq dataset. Figure S9. Expression of prognostic gene signatures in two primary HNC scRNA-Seq datasets. Figure S10. Expression of prognostic gene signatures in four major cell types, as indicated by bulk RNA-Seq-derived transcriptional profiles of flow sorted cells. Figure S11. Correlations of prognostic gene signatures with an epithelial to mesenchymal transition (EMT) transcriptional score within primary HNC malignant cells. Figure S12. UMAPs (Seurat feature plots) showing fibroblast and myofibroblast gene signatures in primary HNCs of the Puram and Stanford primary HNC scRNA-Seq datasets. Figure S13. Expression of lymph node metastasis (LNM) and differentiation-related gene signatures in two primary HNC single cell RNA-Seq datasets (Supplementary to figure 2D). Figure S14. Expression of LNM-associated gene signatures within distinct subpopulations of malignant cells in primary HNCs and patien [file 13073_2023_1236_MOESM1_ESM.pdf]

Supplementary Methods & Results

Investigation of potential confounding factors and effect modifiers in the association of genes with survival

To confirm that the association of genes with survival was not driven by HPV status, a meta-analysis was performed to test the association of all genes with survival in HNC, excluding HPV positive (HPV+ve) cases. HPV status is generally only tested clinically in oropharyngeal cancers (The subset of HNCs occurring in the oropharynx), since HPV is only known to be pathogenic in OPCs and HPV status is associated with survival within OPC specifically[1]. Therefore, to achieve a sufficient sample size for meta-analysis while excluding cases in which HPV was a potential prognostic factor, this meta-analysis excluded both HNCs that were annotated as HPV+ve as well as OPCs that were not annotated for HPV status (Potentially HPV+ve OPCs). This meta-analysis was performed using methods consistent with the main 'all HNC' survival meta-analysis (i.e., the meta-analysis applied to all HNCs irrespective of HPV status), using studies for which survival and HPV information were reported. This meta-analysis included all datasets that had at least 20 primary HNCs with survival and gene expression data, as well as data needed to exclude HPV+ve HNCs (N=13 studies with a combined total of 1,191 HNCs). We then compared survival meta-Z scores derived from this 'HPV-ve' meta-analysis to those derived from the main all HNC meta-analysis, for genes that were associated with survival in the main meta-analysis. Meta-Z scores were strongly correlated between meta-analyses (Pearson R=0.96), indicating that these genes are associated with survival in HPV-ve HNC specifically and that HPV status does not drive their survival associations (**Additional file 1: Figure S4A ii**). We next investigated the possibility that HPV status modifies associations of genes with survival in the TCGA dataset, the study that included

the largest number of HPV+ve cases, and for which HPV status was determined based on detection of HPV RNA, the most accurate indicator[1]. Cox proportional hazard models were applied to test the association of each gene with survival, including an interaction term between gene expression and HPV status. Significant interactions were observed for only 20/1209 (<2%) of survival-associated genes (**Additional file 1: Figure S4A iii)**, indicating that HPV status does not affect the association of genes with survival in most cases. To investigate potential confounding by age or sex, a meta-analysis was performed to test the association of genes with survival adjusting for these variables. This meta-analysis included all datasets that had at least 20 primary HNCs with survival and gene expression data, as well as annotation for age and sex (N=10 studies with a combined total of 1,096 HNCs). For each study dataset separately, multivariate Cox regression models were used to calculate z-scores for association of each gene with survival, adjusting for age and sex. Meta-Z scores were then calculated using Liptak's weighted meta-Z test to combine z-scores across studies, using methods that were otherwise consistent with those applied to the main meta-analysis (i.e., the survival analysis based of univariate Cox regression models). Survival meta-Z scores derived from this meta-analysis were then compared with those derived from the main univariate meta-analysis, which indicated strong consistency between meta-analyses for genes that were associated with survival in the main meta-analysis (Pearson R=0.99, **Additional file 1: Figure S4A iv**). This indicates that the associations of these genes with survival are generally independent of age and sex.

Investigation of potential confounding factors and effect modifiers in the association of genes with lymph node metastasis (LNM) status

To determine if associations of genes with LNM status were confounded by HPV, a meta-analysis was performed to test the association of all genes with LNM status in HNCs excluding HPV positive (HPV+ve) cases. As for the survival gene meta-analysis excluding HPV+ve HNC (See "*Supplementary Methods: Investigation of potential confounding factors and effect modifiers in the association of genes with survival"*), the effect of HPV status was excluded by omitting all HNCs that were annotated as HPV+ve, as well as OPCs that were not annotated for HPV status (Potentially HPV+ve OPVs). This meta-analysis included all datasets that had at least five LNM0 and five LNM+ primary HNCs with gene expression data and data needed to exclude HPV+ve HNCs (N=9 studies with a combined total of 948 HNCs). Using methods consistent with the main LNM gene meta-analysis (that includes all HNCs irrespective of HPV status), random effects models were used to test the association of each gene with LNM status. Meta-Z scores derived from this meta-analysis were highly consistent with those derived from the main 'all HNC' meta-analysis for genes that were associated with LNM status in the main meta-analysis (Pearson R=0.97, **Additional file 1: Figure S4B ii**). This indicates that LNM-associated genes identified by the main analysis are associated with LNM status in HPV-ve HNC specifically (**Additional file 1: Figure S4B ii**). We then investigated the possibility that HPV modifies the association of genes with LNM status in the TCGA dataset. To investigate this, logistic regression models were applied to test the association of each gene with LNM status, including an interaction term between genes and HPV status. Significant interactions with HPV status were not observed for any gene (**Additional file 1: Figure S4B iii**), suggesting that HPV status does not strongly modify the association of genes with LNM status. Importantly, however, it is likely that this analysis was underpowered to detect significant interactions between genes and HPV status due to the small number of HPV+ve HNCs without lymph node metastases (N=13) that could be included. This reflects the low prevalence of HNCs annotated as HPV+ve cases within the available datasets, combined with the more

metastatic nature of HPV+ve HNC relative with HPV-ve HNC[2,3]. To investigate potential confounding by age or sex, a meta-analysis was performed to test the association of genes with LNM status adjusting for these variables. This meta-analysis included all datasets that had at least five LNM0 and five LNM+ primary HNCs with gene expression, age, and sex data (N=13 studies with a combined total of 1,181 HNCs). For each study separately, multivariate logistic regression models were used to calculate z-scores for association of each gene with LNM status, adjusting for age and sex. Meta-Z scores were then calculated using Liptak's weighted meta-Z test to combine z-scores across studies, with weights set to the square roots of dataset sample sizes. We then compared meta-Z scores derived from this meta-analysis with those derived from main LNM gene meta-analysis that was performed using random effects models. Meta-Z scores were highly consistent between these meta-analyses for genes that were associated with LNM status in the main meta-analysis (Pearson R=0.98, **Additional file 1: Figure S4B iv**), indicating that these genes are associated with LNM status independent of age and sex.

Supplementary Discussion

The survival and LNM-associated genes identified by our meta-analyses were associated with these outcomes independent of potential confounding factors including age, and sex, and HPV status, and remained significantly associated in meta-analyses restricted to HPV-ve HNCs. It is not surprising that the genes identified by our meta-analysis remained significantly associated with their respective clinical outcomes after exclusion of HPV+ve cases, given the high prevalence of HPV-ve HNCs in our meta-analyses as well as the inclusion of multiple purely HPV-ve studies. A limitation of this study, however, is that we could not confirm the

association of these genes with clinical outcomes in HPV+ve HNC specifically, due to insufficient patient numbers with HPV status annotation. Moreover, analysis of interactions between genes and HPV status indicated that HPV status did not modify associations of any gene with LNM status, and modified gene-survival associations for fewer than two percent of genes. These analyses were likely underpowered to detect significant interactions between genes and HPV status, especially when considering associations of genes with LNM status, since HPV+ HNCs are disproportionally LNM+[2,3]. Additional patient studies are needed to identify survival and LNM-associated genes in HPV+ve HNC specifically, and might identify gene-HPV interactions, since HPV status is a strong determinant of survival and LNM and defines a transcriptionally distinct subtype[1]. Given the increasing adoption of RNA-Seq, which can be used to accurately infer HPV status, as well as increasing clinical HPV testing in HNC, it will be increasingly possible to identify prognostic pathways in HPV+ve HNC as new studies emerge.

Within the survival gene meta-analysis, Cox regression z-scores indicating the association of genes with survival were largely consistent across studies but inconsistent for some smaller studies, e.g., the Pickering[4] and Bhosale[5] studies. Despite this, z-scores for association of genes with LNM status were consistent between these and other larger studies, suggesting that gene expression data and clinical annotation for these studies was of adequate quality. The inconsistency of survival gene z-scores derived from these studies is therefore likely due to small sample size, such that these studies are underpowered to detect association of genes with survival using Cox regression. Small samples size might have impeded the statistical analysis to a lesser extent for the LNM gene meta-analysis due to the use of random effects models which are non-parametric. This inconsistency of z-scores in some smaller studies highlights

the importance of weighting studies by sample size, which is achieved in our survival meta-analysis using Liptak's weighted meta-Z test, as used previously[6].

Supplementary References

1. Leemans, C. R., Snijders, P. J. F. & Brakenhoff, R. H. The molecular landscape of head and neck cancer. *Nat Rev Cancer* **18**, 269–282 (2018).
2. Bauwens, L. *et al.* Prevalence and distribution of cervical lymph node metastases in HPV-positive and HPV-negative oropharyngeal squamous cell carcinoma. *Radiotherapy and Oncology* (2021) doi:10.1016/j.radonc.2021.01.028.
3. Husain, N. & Neyaz, A. Human papillomavirus associated head and neck squamous cell carcinoma: Controversies and new concepts. *Journal of Oral Biology and Craniofacial Research* **7**, 198 (2017).
4. Pickering, C. R. *et al.* Integrative genomic characterization of oral squamous cell carcinoma identifies frequent somatic drivers. *Cancer Discovery* **3**, 770–781 (2013).
5. Bhosale, P. G. *et al.* Chromosomal Alterations and Gene Expression Changes Associated with the Progression of Leukoplakia to Advanced Gingivobuccal Cancer. *Transl Oncol* **10**, 396–409 (2017).
6. Gentles, A. J. *et al.* The prognostic landscape of genes and infiltrating immune cells across human cancers. *Nature Medicine* **21**, 1–12 (2015).
7. Levine, J. H. *et al.* Data-Driven Phenotypic Dissection of AML Reveals Progenitor-like Cells that Correlate with Prognosis. *Cell* (2015) doi:10.1016/j.cell.2015.05.047.
8. Gentles, A. J. *et al.* The prognostic landscape of genes and infiltrating immune cells across human cancers. *Nature Medicine* (2015) doi:10.1038/nm.3909.

9. Franzén, O., Gan, L.-M. & Björkegren, J. L. M. PanglaoDB: a web server for exploration of mouse and human single-cell RNA sequencing data. *Database* **2019**, 46 (2019).

10. Puram, S. V. *et al.* Single-Cell Transcriptomic Analysis of Primary and Metastatic Tumor Ecosystems in Head and Neck Cancer. *Cell* (2017) doi:10.1016/j.cell.2017.10.044.

11. Gibbons, D. L. & Creighton, C. J. Pan-cancer survey of epithelial–mesenchymal transition markers across the Cancer Genome Atlas. *Developmental Dynamics* **247**, 555–564 (2018).

12. Creighton, C. J., Gibbons, D. L. & Kurie, J. M. The role of epithelial–mesenchymal transition programming in invasion and metastasis: a clinical perspective. *Cancer Management and Research* **5**, 187–195 (2013).

13. Franzén, O., Gan, L.-M. & Björkegren, J. L. M. PanglaoDB: a web server for exploration of mouse and human single-cell RNA sequencing data. *Database* **2019**, 46 (2019).

14. Gulati, G. S. *et al.* Single-cell transcriptional diversity is a hallmark of developmental potential. *Science* (2020) doi:10.1126/science.aax0249.

15. Assou, S. *et al.* A meta-analysis of human embryonic stem cells transcriptome integrated into a web-based expression atlas. *Stem cells (Dayton, Ohio)* **25**, 961–73 (2007).

16. Puram, S. V. *et al.* Single-Cell Transcriptomic Analysis of Primary and Metastatic Tumor Ecosystems in Head and Neck Cancer. *Cell* (2017) doi:10.1016/j.cell.2017.10.044.

17. Assou, S. *et al.* A meta-analysis of human embryonic stem cells transcriptome integrated into a web-based expression atlas. *Stem cells (Dayton, Ohio)* **25**, 961–73 (2007).

18. Gulati, G. S. *et al.* Single-cell transcriptional diversity is a hallmark of developmental potential. *Science* (2020) doi:10.1126/science.aax0249.

Supplementary Figure Legends

Figure S1. FlowJo contour plots illustrating the gating strategy that was used to isolate four cell types from head and neck cancer tumors using fluorescence-activated cell sorting (FACS): Cells were analyzed using FlowJo V. 10.6.1 and first gated on single cell size using FSC width and height and cell granularity using SSC width and height. Live cells were gated using the DAPI stain. From the live cell gate, the leukocyte group (Immune cells) in FITC and endothelial group in PE were used to separate out CD3+CD31 leukocytes from CD3-CD31 endothelial cells. Leukocyte and endothelial negative populations were used to gate further for fibroblasts in APC and the malignant unstained (Leukocyte, endothelial and fibroblast negative) group. These contour plots illustrate gating for one participant (Participant ID: 7228) as a representation of the gating strategy applied to all samples.

Figure S2. Unsupervised clustering of survival and LNM-associated genes based on co-expression: Unsupervised clustering of A) survival-associated and B) LNM-associated genes based on their co-expression in primary HNC populations. Ai) Uniform Manifold Approximation and Projection (UMAP) illustrating clusters of survival-associated genes (Points); these clusters were identified by applying Phenograph[7] to gene expression data for 958 survival-associated genes in 1,642 primary head and neck cancers, which were combined from 20 patient studies. These 958 genes represent the subset of all survival associated genes (n=1,209) for which data was available in at least 80% of the curated patient studies. Point colors indicate gene clusters, while shapes indicating the direction of association with survival. Aii) Box plots of survival meta-z scores for all survival-associated genes (Points), with genes stratified by unsupervised cluster membership (i.e., the survival gene clusters shown in *Ai*). Meta-z scores indicate the significance and direction of the association with survival of each

gene. Red and blue points (With gene symbols) highlight the ten most adversely prognostic and the ten most favorably prognostic genes, respectively, based on their meta-z scores. The grey box “No cluster” category represents survival-associated genes to which unsupervised clustering was not applied, as these genes were represented in fewer than 80% of datasets. Bi) UMAP illustrating clusters of LNM-associated genes (Points), which were identified by applying Phenograph to gene expression data for 742 LNM-associated genes in 1,642 primary head and neck cancers that were derived from 20 patient studies. These 742 genes represent the subset of all LNM associated genes (n=877) for which data was available in at least 80% of the curated patient studies. Point colors indicate gene clusters, while shapes indicating the direction of association with LNM. Bii) Box plots of LNM meta-z scores for all LNM-associated genes (Points), with genes stratified by unsupervised cluster membership (i.e., the LNM gene clusters shown in *Bi*). Meta-z scores indicate the significance and direction of the association with LNM of each gene. Red and blue points (With gene symbols) highlight the ten genes with the strongest positive (Pro-LNM) and negative (Anti-LNM) association with LNM, respectively. The grey box “No cluster” category represents LNM-associated gene to which unsupervised clustering was not applied, as these genes were represented in fewer than 80% of datasets. To improve visualization, Y axes of boxplots are split to exclude the ‘non-significant’ meta-z score range between -3.09 and 3.09.

Figure S3. Pan-cancer survival meta-z scores of genes that were associated with survival and lymph node metastasis (LNM) in head and neck cancer (HNC): For gene signatures that were associated with A) survival and B) LNM in HNC (Based on our meta-analyses within the current study), the box plots indicate PRECOG(Gentles et al. 2015) pan-cancer survival z-scores, which indicate the association of each gene with overall survival in cancer overall. Pan-cancer survival z-scores were calculated as part of our previously reported meta-analysis of

survival-associated genes[8]. Survival-associated signatures (A) include all genes that were negatively (Anti-survival) and positively (Pro-survival) associated with survival in HNC, as well as genes within survival gene clusters (S1-6). LNM-associated gene signatures (B) include all genes that were negatively (Anti-LNM) and positively (Pro-LNM) associated with LNM in HNC, as well as genes within LNM gene clusters (L1-6). Red and blue points (With gene symbols) highlight genes that were within the top hundred most adversely prognostic (Red) and the 100 most favorably prognostic (Blue) in cancer overall, based on pan-cancer meta-z scores. Horizontal dashed lines indicate the Y -axis position of genes with a pan-cancer meta-z score of zero, representing genes that display no associated with survival in cancer overall.

Figure S4. Independence of prognostic signatures from potential confounding factors. Analyses performed to investigate the potential confounding effect of human papillomavirus (HPV) status, age, and sex, on association of genes associated with A) patient survival and B) lymph node metastasis (LNM) status. A) independence of survival-associated gene from confounding factors. I) Heatmap showing z-scores for the association of survival signature expression scores with survival in COX proportional hazard regression models adjusted for HPV status. Survival signatures shown in *i* include all genes that were negatively (Anti-survival) and positively (Pro-survival) associated with survival, and genes within survival gene clusters (S1-6). Survival signature expression scores were calculated as the mean expression of genes within each survival gene signature (i.e., set of genes). Z-scores were calculated using cox regression models to test for association of each signature expression score with survival, adjusting for HPV status, in all studies (N=4) for which both survival and HPV data were available. Row names represent meta-z scores indicating the overall association of gene signatures with survival across studies. Meta-z scores were calculated using Liptak's weighted meta-z test to combine z-scores across studies, weighted by study sample size. ii) Smoothed

scatter plot showing consistency of survival gene meta-z scores when excluding HPV positive (HPV+) HNCs. The scatter plot compares survival meta-z scores derived from two separate meta-analyses including (X-axis) a meta-analysis of gene-survival associations in HNCs excluding HPV+ oropharyngeal cancers (OPCs) and (Y-axis) a meta-analysis of gene-survival associations in all HNCs irrespective of HPV status. Points represent genes associated with survival in the main "All HNC" meta-analysis. The regression lines (Red dashed lines) and Pearson correlation coefficient (R) indicate the correlation between meta-z scores derived from the two meta-analyses. iii) Analysis of interaction effect of HPV status on association of genes with survival. Box plot of negative log ten p-values for interactions between genes and HPV status in cox proportional hazard models testing associations of genes (Points) with overall survival in the TCGA study. Genes are stratified by unsupervised gene cluster to investigate potential modifying effects of HPV status on survival associations for genes within each cluster. The red dashed line indicates the statistical significance threshold (Equivalent to p=0.05). iv) Consistency of survival meta-z scores when adjusting for age and sex. Smoothed scatter plot comparing meta-z scores derived from two separate meta-analyses, one that tested the association of genes (points) with survival using univariate Cox proportional hazard regression models (Y axis), the other that tested the association of genes with survival using multivariate models Cox proportional hazard regression models adjusted for patient age and sex (X-axis). Meta-z scores were calculated using Liptak's weighted meta-z test to combine z-scores across studies. Per-gene survival z-scores were calculated for each study using either univariate models or multivariate models adjusted for age and sex. Points represent genes that were associated with survival in the main univariate meta-analysis. Regression lines (Red dashed lines) and Pearson correlation coefficients (R) indicate the correlation between meta-z scores derived from the two meta-analyses. B) independence of LNM-associated gene from confounding factors. i) Heatmap showing z-scores for associations of LNM signature

expression scores with LNM status in logistic regression models adjusted for HPV status. LNM signatures shown in *B* include all genes negatively (Anti-LNM) and positively (Pro-LNM) associated with LNM, and genes within LNM gene clusters (L1-6). LNM signature expression scores were calculated as mean expression of genes within each LNM gene signature (i.e., set of genes). Z-scores were calculated using logistic regression models testing associations of each LNM signature score with LNM status, adjusting for HPV status, in all studies (N=6) for which both LNM and HPV data were available. Row names represent meta-z scores indicating the overall association of the gene signatures with LNM status across studies. Meta-z scores were calculated using Liptak's weighted meta-z test to combine z-scores across studies, weighted by study sample size. ii) Smoothed scatter plot showing consistency of LNM gene meta-z scores when excluding HPV positive (HPV+) HNCs. The scatter plot compares LNM meta-z scores derived from two separate meta-analyses including (X-axis) a meta-analysis of gene-LNM associations in HNCs excluding HPV+ oropharyngeal cancers (OPCs) and (Y-axis) a meta-analysis of gene-LNM associations in all HNCs irrespective of HPV status. Points represent genes associated with LNM in the main "All HNC" meta-analysis. The regression lines (Red dashed lines) and Pearson correlation coefficient (R) indicate the correlation between meta-z scores derived from the two meta-analyses. Regression lines (Red dashed lines) and Pearson correlation coefficients (R) indicate the correlation of meta-z scores between the two meta-analyses. Meta-Z scores are shown for genes that were associated with LNM status in the main "All HNC" meta-analysis. iii) Analysis of interaction effect of HPV status on association of genes with LNM status. Box plot of negative log ten p-values for interactions between genes and HPV status, in logistic regression models testing associations of genes (Points) with LNM status in the TCGA study. Genes are stratified by unsupervised LNM gene cluster to investigate potential modifying effects of HPV status on LNM associations of genes within each cluster. The red dashed line indicates the statistical significance threshold

(Equivalent to p=0.05). iv) Consistency of LNM gene meta-z scores when adjusting for age and sex. Smoothed scatter plot comparing meta-z scores derived from two separate meta-analyses, one that tested the association of genes (points) with LNM status using univariate logistic regression models (Y-axis), the other that tested the association of genes with LNM status using multivariate logistic regression models adjusted for patient age and sex (X-axis). Meta-Z scores were calculated using Liptak's weighted meta-z test to combine z-scores across studies. Per-gene LNM z-scores were calculated for each study using either univariate models or multivariate models adjusted for age and sex. Points represent genes that were associated with LNM in the main univariate meta-analysis. Regression lines (Red dashed lines) and Pearson correlation coefficients (R) indicate the correlation between meta-z scores derived from the two meta-analyses.

Figure S5. Meta-analysis-based identification of genes associated with tumor grade in HNC: Heatmap showing linear regression z-scores for association of genes (Rows) with tumor grade (i.e., level of pathological differentiation) based on a meta-analysis that included 13 HNC gene expression studies (Columns). Z-scores indicate the significance of association of genes with grade, where grade was reported either using a numeric grading system or as the level of differentiation upon histological analysis (Well, moderate, poor). Genes are ordered by meta-z score (Right sidebar), indicating the overall association of each gene with grade across studies. The heatmap shows all genes (N=5,068) that were significantly associated with grade, including 3,108 genes that were positively associated (Pro-grade, meta-z ≥3.09) and 1,960 that were negatively associated (Anti-grade, meta-z ≤-3.09).

Figure S6. Overlap of genes between LNM gene clusters and survival gene clusters: Bar plot showing the number of genes within each LNM cluster (L1-L6) that overlapped with each

survival gene cluster (S1-S6). The asterisk indicates the statistical significance of the overlap between genes within LNM gene cluster L2 and genes within survival gene cluster S6. ***: Hypergeometric test p-value <0.001.

Figure S7. UMAP representations of primary HNCs within the Stanford scRNA-Seq dataset: A) UMAPs (Dimensionality plots) showing unsupervised cell clusters, cell types, and cell cycle phase of all cells within primary HNCs, as well as the patient sample from which each cell derived (N=5 primary HNCs, each derived from a separate patient). B) UMAPs (Seurat feature plots) showing expression of cell type marker gene signatures that were derived from the PanglaoDB database[9] and that were used to assign cell types. Each UMAP shows an expression score for a cell type marker signature (i.e., set of cell type marker genes), representing the mean expression (Normalized counts) of all genes within the signature. Points represent cells with the color gradient indicating the gene expression score.

Figure S8. UMAP representations of the Puram scRNA-Seq dataset: A) UMAPs (Dimensionality plots) showing unsupervised cell clusters, cell types, and cell cycle phase of all cells within primary HNCs, as well as the patient sample from which each cell derived (N=9 primary HNCs, each derived from a separate patient). Cell type labels were previously assigned by Puram et *al.*[10] and were confirmed using cell type marker gene signatures shown in *B*. B) UMAPs (Seurat feature plots) showing expression of cell type marker gene signatures that were derived from the PanglaoDB database[9] and that were used to confirm cell type annotations. Each UMAP shows an expression score for a cell type marker signature (i.e., set of cell type marker genes), representing the mean expression (Normalized counts) of all genes within the signature. Points represent cells with the color gradient indicating the gene expression score.

Figure S9. Expression of prognostic gene signatures in two primary HNC scRNA-Seq datasets: Plots illustrating expression of A) survival and B) LNM-associated gene signatures in primary HNCs of two scRNA-Seq datasets, including the Puram and Stanford datasets. Equivalent figure panels are shown for survival and LNM-associated genes. Survival-associated genes include all genes that were negatively (Anti-survival) and positively (Pro-survival) associated with survival, genes within survival gene clusters (S1-6). LNM-associated genes include all genes that were negatively (Anti-LNM) and positively (Pro-LNM) associated with lymph node metastasis (LNM), and genes within LNM gene clusters (L1-6). i) Violin plots showing expression scores of prognostic gene signatures in two scRNA-Seq datasets with cells (Points) stratified by cell type. Expression scores represent the scaled mean expression (Normalized counts) of all genes within prognostic signature (i.e., set of survival or LNM-associated genes). ii) UMAPs (Seurat feature plots) showing expression scores of prognostic gene signatures in two primary HNC scRNA-Seq datasets, including the Puram and Stanford datasets. Figures S6 and S7 include corresponding UMAPs that indicate cell types and phenotypes for the Stanford and Puram datasets, respectively.

Figure S10. Expression of prognostic gene signatures in four major cell types, as indicated by bulk RNA-Seq-derived transcriptional profiles of flow sorted cells: A) Violin plots showing expression scores of prognostic gene signatures (Y axes) in bulk gene expression profiles of cells that were sorted from primary HNCs (N=15) using fluorescence activated cell sorting (FACS). Points represent samples/gene expression profiles and are stratified by cell type. Each sample represents the bulk gene expression profile of cells that were flow-sorted from one primary HNC. Samples include endothelial cells that were isolated from 12 HNCs, fibroblasts from ten HNCs, leukocytes from 15 HNCs, and malignant cells from 13 HNCs. Expression scores represent mean of expression (Log2 normalized counts) of genes within each prognostic

signature. Prognostic signatures include all genes that were negatively (Anti-survival) and positively (Pro-survival) associated with survival, genes within survival gene clusters (S1-6), all genes that were negatively (Anti-LNM) and positively (Pro-LNM) associated with LNM, and genes within LNM gene clusters (L1-6). B) Heatmap of CIBERSORTx-inferred cell fractions within samples of the Stanford bulk RNA-Seq dataset, which was generated by applying bulk RNA-Seq to flow-sorted cells. Cell fractions were estimated using CIBERSORTx in combination with a signature matrix derived from primary HNCs of the Puram scRNA-Seq dataset.

Figure S11. Correlations of prognostic gene signatures with an epithelial to mesenchymal transition (EMT) transcriptional score within primary HNC malignant cells: A) UMAPs (Seurat feature plots) showing EMT score as well as expression scores of mesenchymal and epithelial marker genes in two primary HNC scRNA-Seq datasets including the i) Puram and ii) Stanford datasets. Points represent malignant cells. EMT score was calculated as the sum of normalized counts of mesenchymal genes (*VIM*, *CDH2*, *FOXC2*, *SNAI1*, *SNAI2*, *TWIST1*, *GSC*, *FN1*, *ITGB6*, *MMP2*, *MMP3*, *MMP9*, and *SOX10)* minus the sum of normalized counts of epithelial genes (*CDH1*, *DSP*, and *TJP1*), as previously described[11,12]. Separate expression scores of the mesenchymal (EMT mesenchymal) and epithelial (EMT epithelial) genes are shown in the additional UMAPs, with expression scores representing the mean normalized counts of all mesenchymal and epithelial genes, respectively. B) Correlations of prognostic signatures with EMT score in malignant cells of primary HNCs within the Puram and Stanford scRNA-Seq datasets. Points illustrate associations between EMT score and expression scores of each prognostic gene signature within malignant cells. Pearson correlation coefficients (r) is represented by the point color gradient, while point sizes represent negative log ten p-values (Linear regression). Prognostic signatures include all genes that were negatively (Anti-

survival) and positively (Pro-survival) associated with survival, genes within survival gene clusters (S1-6), all genes that were negatively (Anti-LNM) and positively (Pro-LNM) associated with LNM, and genes within LNM gene clusters (L1-6). Expression scores were calculated for each prognostic signature (i.e., set of prognostic genes) as the mean of expression (Normalized counts) of all genes within the signature.

Figure S12. UMAPs (Seurat feature plots) showing fibroblast and myofibroblast gene signatures in primary HNCs of the Puram and Stanford primary HNC scRNA-Seq datasets: Gene signature expression scores are shown for three gene signatures including fibroblast and myofibroblast marker genes that were accessed from the PanglaoDB database[13], a set of marker genes that are highly expressed in CAF1 fibroblasts, as reported by Puram *et al*. Also shown are UMAPs illustrating expression (Normalized counts) of three individual myofibroblast marker genes (*ACTA2*, *MYLK*, and *MYL9*).

Figure S13. Expression of lymph node metastasis (LNM) and differentiation-related gene signatures in two primary HNC single cell RNA-Seq datasets (Supplementary to figure 2D): Uniform Manifold Approximation and Projections (UMAPs) illustrating cell phenotypes, as well as the expression of LNM and differentiation-associated gene signatures, within primary tumors of the A) Stanford, and B) Puram scRNA-Seq datasets. Points represent cells. Equivalent UMAPs are shown for both scRNA-Seq datasets for completeness, however some of these UMAPs are also shown for the Puram dataset in *Figure 2D*. i) UMAPs showing cell phenotypes, including cell type, unsupervised cell cluster, cell cycle phase, and level of transcriptional diversity within malignant cells. Transcriptional diversity, an indicator of pluripotency, is represented on the UMAP as a CytoTRACE[14] score. ii) UMAPs (Seurat feature plots) showing expression scores of LNM gene signatures. LNM-associated gene signatures

include all genes that were negatively (Anti-LNM) and positively (Pro-LNM) associated with LNM in our meta-analysis, as well as genes within anti-LNM cluster L2 and pro-LNM cluster L4, the largest clusters of LNM-associated genes. iii) UMAPs (Seurat feature plots) showing expression scores of gene signatures related to epithelial differentiation and stemness. Grade-associated gene signatures include all genes that were positively (Pro-grade) and negatively (Anti-grade) associated with tumor grade in our meta-analysis. Embryonic stem cell (ESC) marker genes represent genes specifically expressed in ESCs[15]. Epithelial differentiation markers represent genes identified as part of an epithelial differentiation program in HNC (Referred to as “Epi dif. 1”) based on the original analysis of the Puram dataset[10].

Figure S14. Expression of LNM-associated gene signatures within distinct subpopulations of malignant cells in primary HNCs and patient-matched lymph node metastases (LNMs). Figures show expression of LNM gene signatures in two HNC scRNA-Seq datasets including the A) Puram and B) Stanford datasets. Equivalent figures are shown for the Puram and Stanford scRNA-Seq datasets in *A* and *B*, respectively.

i) Uniform Manifold Approximation and Projections (UMAPs) showing expression scores of LNM gene signatures. These signatures include all genes that were negatively (Anti-LNM) and positively (Pro-LNM) associated with LNM status in our meta-analysis, as well as pro-LNM cluster L4, the largest unsupervised cluster of pro-LNM genes. Expression scores are calculated for each cell as the scaled mean expression (Normalized counts) of genes within each signature (i.e., set of LNM-associated genes). ii) UMAPs corresponding to those shown in *i*, showing cell phenotypes including unsupervised cell cluster, cell type, and cell cycle phase. iii) Scatter plots of expression scores of anti-LNM genes (X-axes) and pro-LNM genes (Y-axes) in malignant cells (Points), with cells stratified by tumor type (Primary HNC or LNM). Point colors indicate unsupervised cell clusters, corresponding to those shown in the UMAP shown

in *ii*. Dashed horizontal and vertical lines at zero mark the mean levels of anti-LNM and pro-LNM expression scores, separating cells into quadrants based on expression of anti-LNM and pro-LNM genes and illustrating their mutually exclusive expression pattern. iv) Density plots of Anti-LNM gene signature scores in malignant cells of primary HNCs and patient matched LNMs. Density plots represent the same data shown in scatter plots in *iii*, and are plotted on corresponding X-axes. Separate curves are shown for cells within each unsupervised cell cluster (indicated by color), corresponding to clusters shown in UMAPs in *i*, and scatter plots in *iii*. Vertical dashed lines indicate mean levels of anti-LNM expression score within each cell cluster, illustrating differences of anti-LNM gene expression between LNMs and primary HNCs within clusters. V) Scatter plots equivalent to those shown in iii, with color gradients indicating scaled mean expression of tumor plasticity gene signatures within malignant cells. Tumor plasticity signatures include: Epithelial differentiation markers: Genes identified as part of an epithelial differentiation-related transcriptional program in HNC (Referred to as "Epi dif. 1") based on the original analysis of the Puram dataset[16]; ESC markers: genes specifically expressed in embryonic stem cells (ESCs)[17], tumor grade-associated gene signatures; and EMT mesenchymal genes: mesenchymal genes used to calculated epithelial to mesenchymal (EMT) scores in this and previous studies[11,12]. Also shown is a scatter plot colored by CytoTRACE score, a measure of transcriptional diversity and stemness[18].

Figure S1

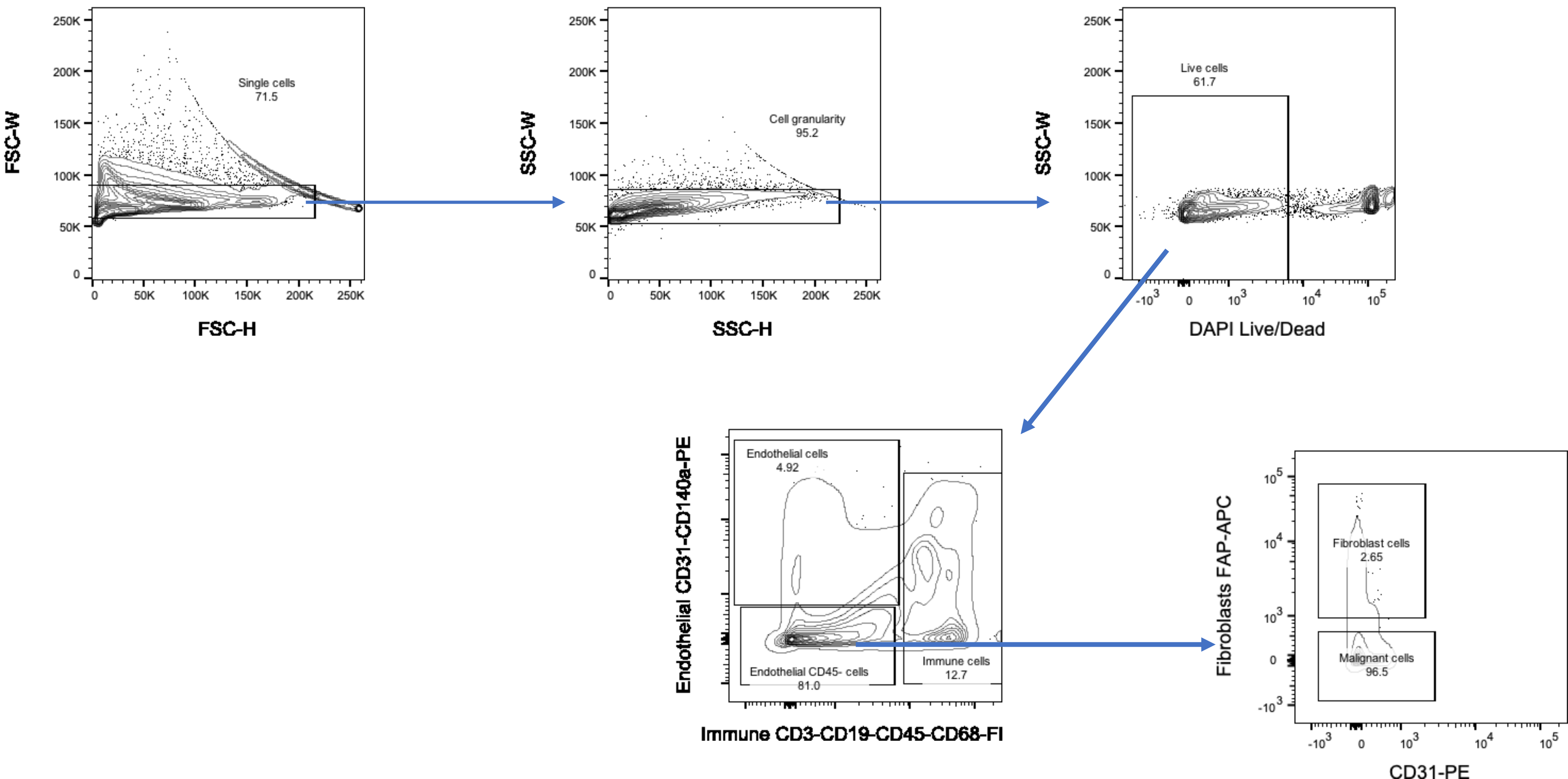

Single cells
71.5
FSC-W
FSC-H
Cell granularity
95.2
SSC-W
SSC-H
Live cells
61.7
DAPI Live/Dead
Endothelial cells
4.92
Endothelial CD45- cells
81.0
Immune cells
12.7
Endothelial CD31-CD140a-PE
Immune CD3-CD19-CD45-CD68-FI
Fibroblast cells
2.65
Malignant cells
96.5
Fibroblasts FAP-APC
CD31-PE

Figure S2

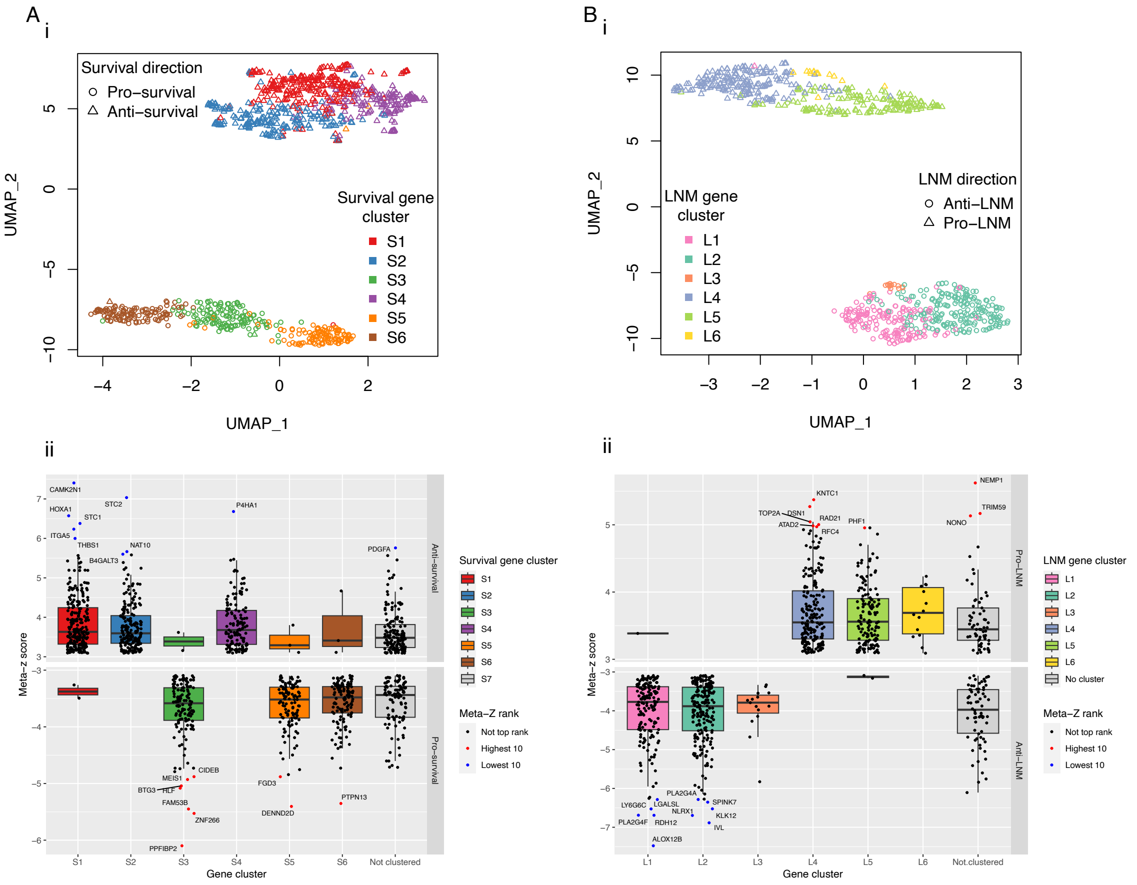

A
i
Survival direction
Pro–survival
Anti–survival
Survival gene cluster
S1
S2
S3
S4
S5
S6
UMAP_1
UMAP_2
B
i
LNM direction
Anti–LNM
Pro–LNM
LNM gene cluster
L1
L2
L3
L4
L5
L6
UMAP_1
UMAP_2
ii
CAMK2N1
HOXA1
STC2
STC1
ITGA5
THBS1
NAT10
B4GALT3
P4HA1
PDGFA
Anti–survival
Pro–survival
CIDEB
MEIS1
BTG3
HLF
FAM53B
ZNF266
PPFIBP2
FGD3
DENND2D
PTPN13
Meta–z score
Gene cluster
S1
S2
S3
S4
S5
S6
Not clustered
Survival gene cluster
S1
S2
S3
S4
S5
S6
S7
Meta–Z rank
Not top rank
Highest 10
Lowest 10
ii
KNTC1
TOP2A
DSN1
RAD21
ATAD2
RFC4
PHF1
NEMP1
TRIM59
NONO
Pro–LNM
Anti–LNM
PLA2G4A
LY6G6C
LGALSL
SPINK7
NLRX1
KLK12
PLA2G4F
RDH12
IVL
ALOX12B
Meta–z score
Gene cluster
L1
L2
L3
L4
L5
L6
Not.clustered
LNM gene cluster
L1
L2
L3
L4
L5
L6
No cluster
Meta–Z rank
Not top rank
Highest 10
Lowest 10

Figure S3

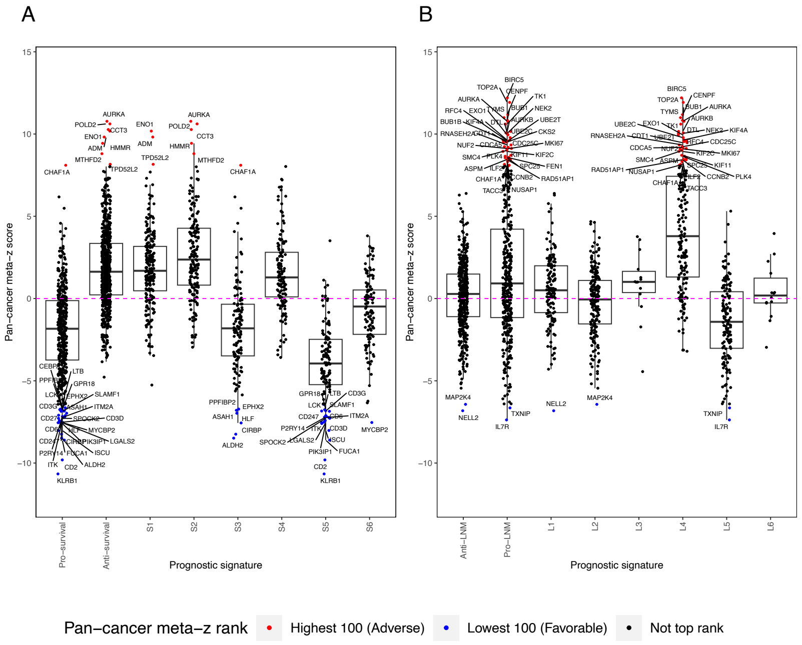

A
B
Pan−cancer meta−z score
Prognostic signature
Pro−survival
Anti−survival
S1
S2
S3
S4
S5
S6
Anti−LNM
Pro−LNM
L1
L2
L3
L4
L5
L6
Pan−cancer meta−z rank
Highest 100 (Adverse)
Lowest 100 (Favorable)
Not top rank

Figure S4

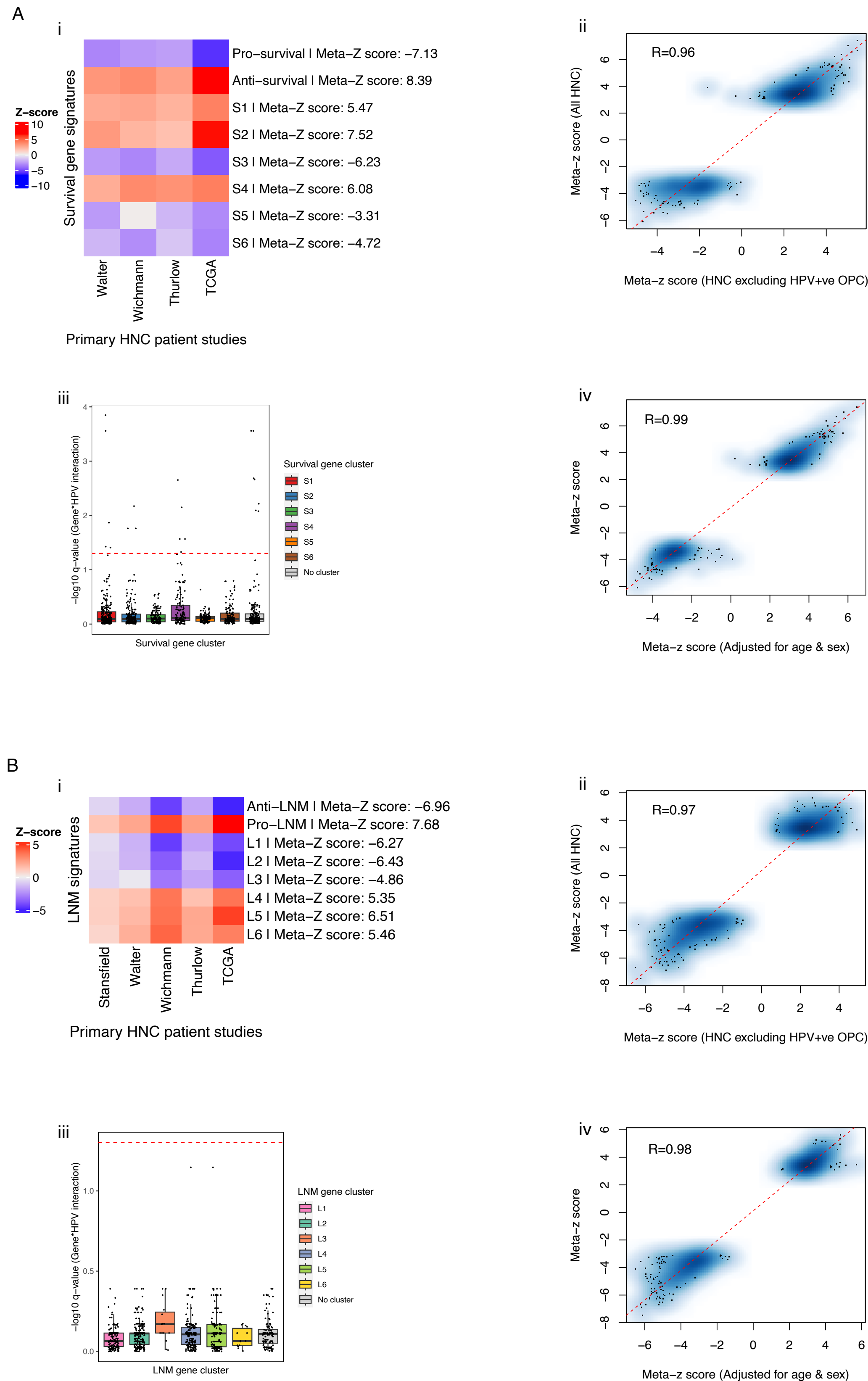
A
i
Pro−survival | Meta−Z score: −7.13
Anti−survival | Meta−Z score: 8.39
S1 | Meta−Z score: 5.47
S2 | Meta−Z score: 7.52
S3 | Meta−Z score: −6.23
S4 | Meta−Z score: 6.08
S5 | Meta−Z score: −3.31
S6 | Meta−Z score: −4.72
Z−score
10
5
0
−5
−10
Survival gene signatures
Walter
Wichmann
Thurlow
TCGA
Primary HNC patient studies
ii
R=0.96
Meta−z score (All HNC)
Meta−z score (HNC excluding HPV+ve OPC)
iii
−log10 q−value (Gene*HPV interaction)
Survival gene cluster
S1
S2
S3
S4
S5
S6
No cluster
Survival gene cluster
iv
R=0.99
Meta−z score
Meta−z score (Adjusted for age & sex)
B
i
Anti−LNM | Meta−Z score: −6.96
Pro−LNM | Meta−Z score: 7.68
L1 | Meta−Z score: −6.27
L2 | Meta−Z score: −6.43
L3 | Meta−Z score: −4.86
L4 | Meta−Z score: 5.35
L5 | Meta−Z score: 6.51
L6 | Meta−Z score: 5.46
Z−score
5
0
−5
LNM signatures
Stansfield
Walter
Wichmann
Thurlow
TCGA
Primary HNC patient studies
ii
R=0.97
Meta−z score (All HNC)
Meta−z score (HNC excluding HPV+ve OPC)
iii
−log10 q−value (Gene*HPV interaction)
LNM gene cluster
L1
L2
L3
L4
L5
L6
No cluster
LNM gene cluster
iv
R=0.98
Meta−z score
Meta−z score (Adjusted for age & sex)

Figure S5

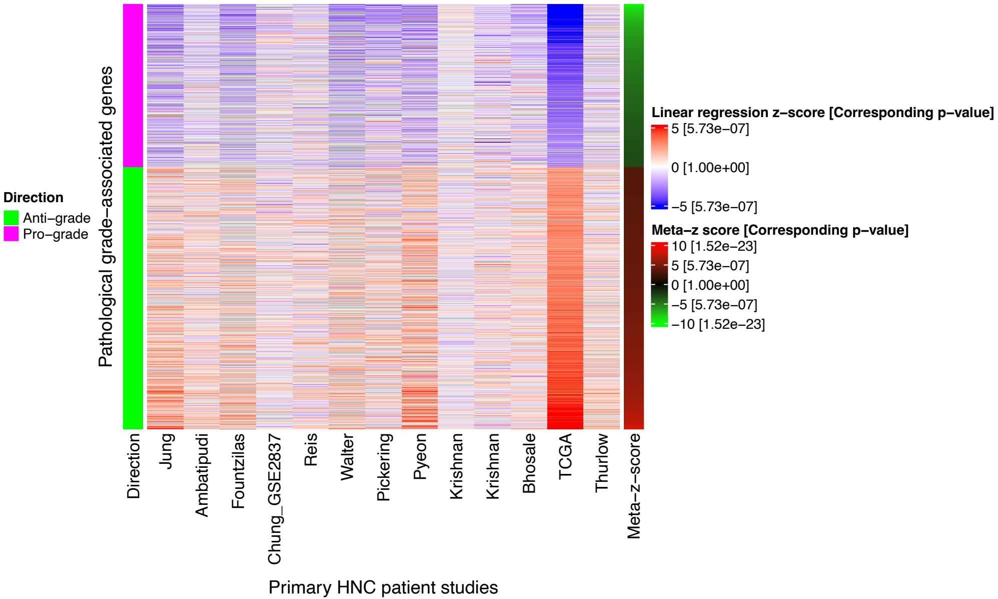
Pathological grade–associated genes
Direction
Anti–grade
Pro–grade
Linear regression z–score [Corresponding p–value]
5 [5.73e–07]
0 [1.00e+00]
–5 [5.73e–07]
Meta–z score [Corresponding p–value]
10 [1.52e–23]
5 [5.73e–07]
0 [1.00e+00]
–5 [5.73e–07]
–10 [1.52e–23]
Direction
Jung
Ambatipudi
Fountzilas
Chung_GSE2837
Reis
Walter
Pickering
Pyeon
Krishnan
Krishnan
Bhosale
TCGA
Thurlow
Meta–z–score
Primary HNC patient studies

Figure S6

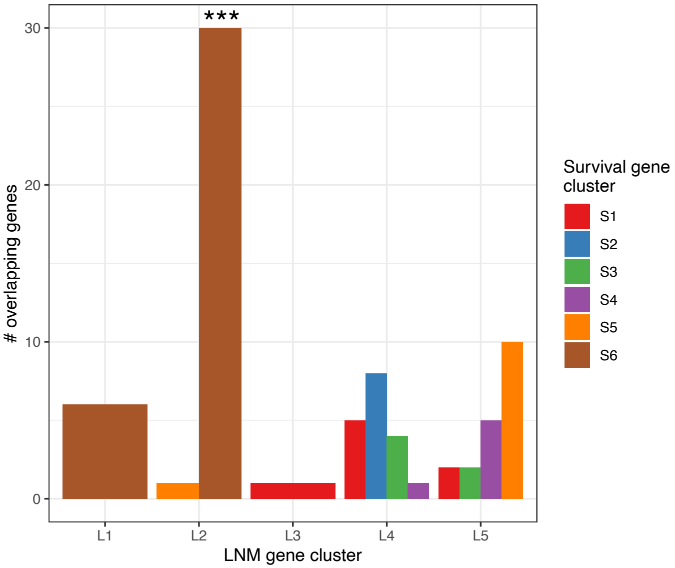

***
30
20
10
0
overlapping genes
L1
L2
L3
L4
L5
LNM gene cluster
Survival gene cluster
S1
S2
S3
S4
S5
S6

Figure S7

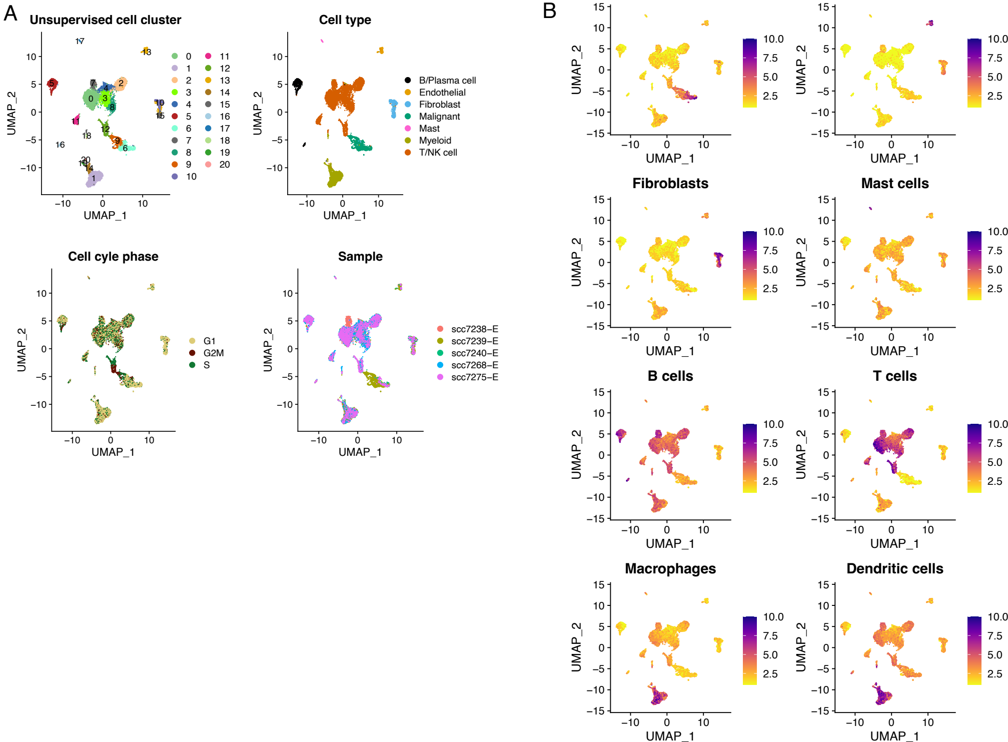
A
Unsupervised cell cluster
Cell type
B/Plasma cell
Endothelial
Fibroblast
Malignant
Mast
Myeloid
T/NK cell
Cell cyle phase
G1
G2M
S
Sample
scc7238–E
scc7239–E
scc7240–E
scc7268–E
scc7275–E
UMAP_1
UMAP_2
B
Epithelial cells
Endothelial cells
Fibroblasts
Mast cells
B cells
T cells
Macrophages
Dendritic cells

Figure S8

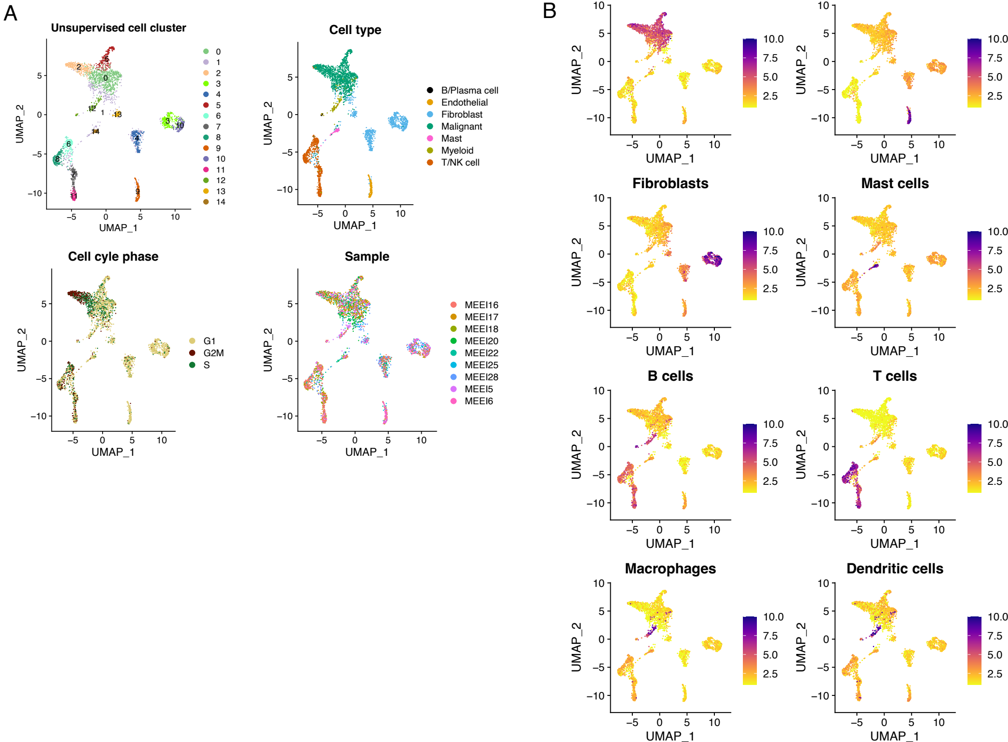

A
Unsupervised cell cluster
UMAP_1
UMAP_2
0
1
2
3
4
5
6
7
8
9
10
11
12
13
14
Cell type
B/Plasma cell
Endothelial
Fibroblast
Malignant
Mast
Myeloid
T/NK cell
Cell cyle phase
G1
G2M
S
Sample
MEEI16
MEEI17
MEEI18
MEEI20
MEEI22
MEEI25
MEEI28
MEEI5
MEEI6
B
Epithelial cells
Endothelial cells
Fibroblasts
Mast cells
B cells
T cells
Macrophages
Dendritic cells
10.0
7.5
5.0
2.5

Figure S9

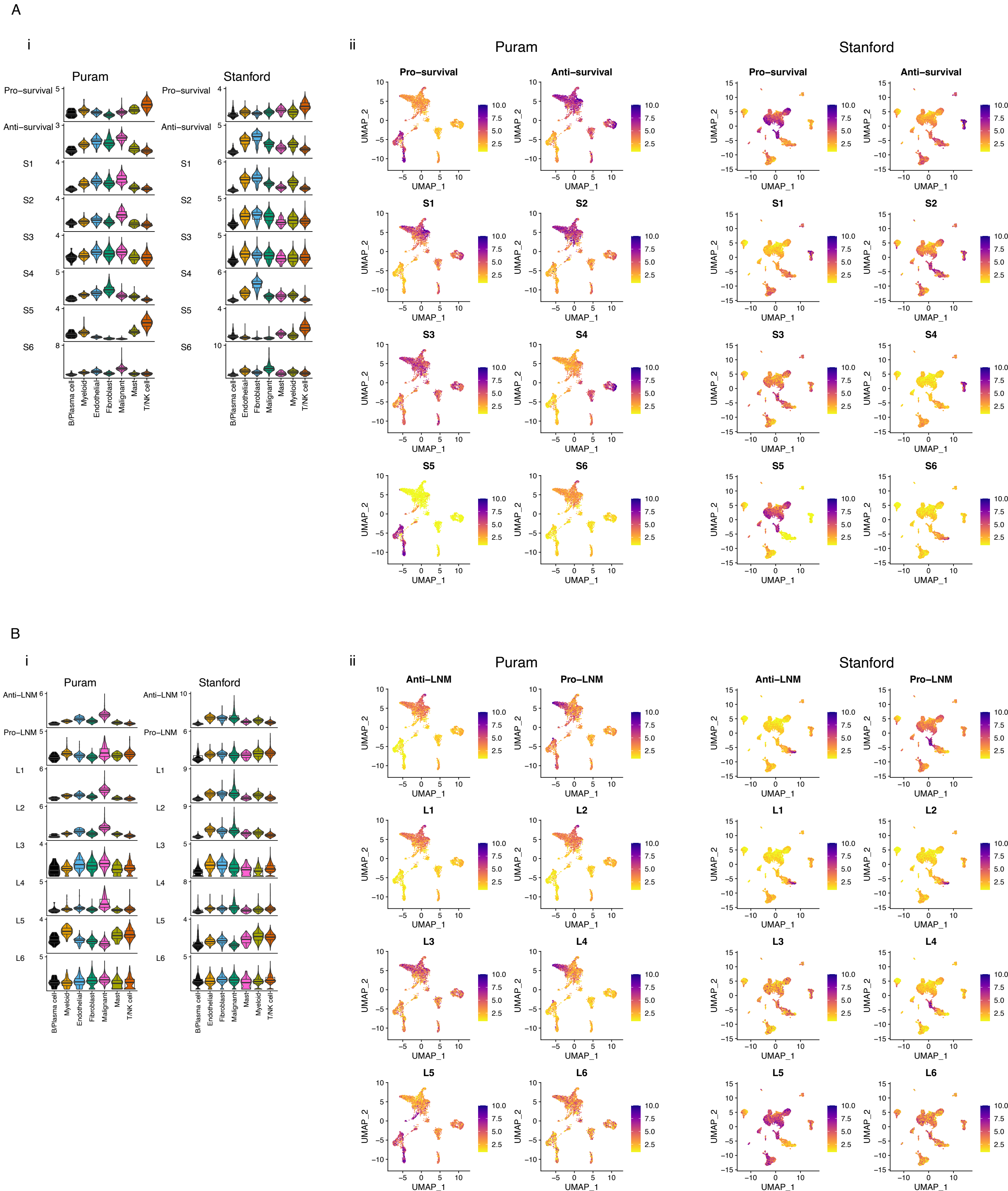
A
i
Puram
Stanford
Pro–survival
Anti–survival
S1
S2
S3
S4
S5
S6
B/Plasma cell
Myeloid
Endothelial
Fibroblast
Malignant
Mast
T/NK cell
ii
Puram
Stanford
Pro–survival
Anti–survival
S1
S2
S3
S4
S5
S6
UMAP_1
UMAP_2
10.0
7.5
5.0
2.5
B
i
Puram
Stanford
Anti–LNM
Pro–LNM
L1
L2
L3
L4
L5
L6
ii
Puram
Stanford
Anti–LNM
Pro–LNM
L1
L2
L3
L4
L5
L6
UMAP_1
UMAP_2

Figure S10

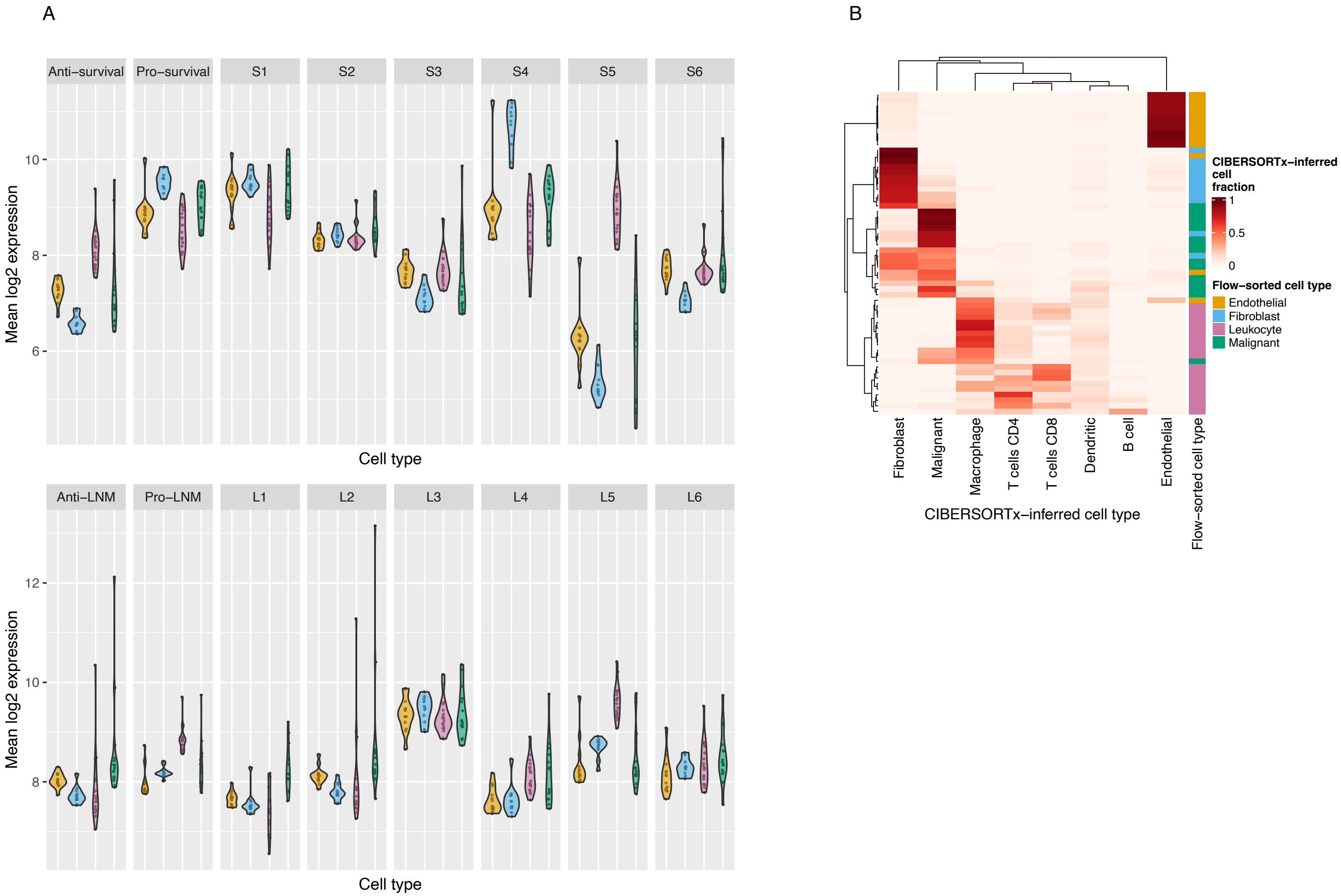

A
Anti–survival
Pro–survival
S1
S2
S3
S4
S5
S6
Mean log2 expression
10
8
6
Cell type
Anti–LNM
Pro–LNM
L1
L2
L3
L4
L5
L6
12
10
8
Cell type
B
CIBERSORTx–inferred cell fraction
1
0.5
0
Flow–sorted cell type
Endothelial
Fibroblast
Leukocyte
Malignant
Fibroblast
Malignant
Macrophage
T cells CD4
T cells CD8
Dendritic
B cell
Endothelial
Flow–sorted cell type
CIBERSORTx–inferred cell type

Figure S11

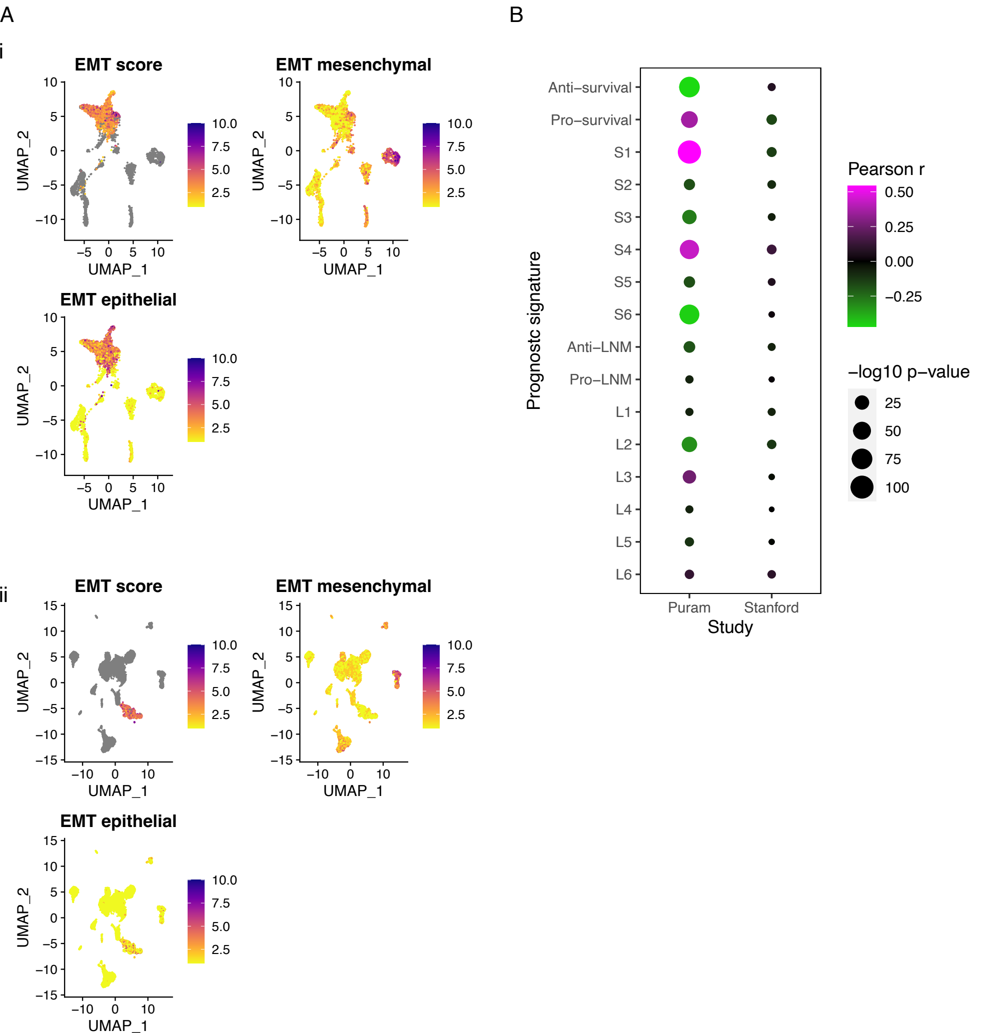
A
i
EMT score
EMT mesenchymal
EMT epithelial
UMAP_1
UMAP_2
10.0
7.5
5.0
2.5
ii
EMT score
EMT mesenchymal
EMT epithelial
B
Anti–survival
Pro–survival
S1
S2
S3
S4
S5
S6
Anti–LNM
Pro–LNM
L1
L2
L3
L4
L5
L6
Prognostc signature
Puram
Stanford
Study
Pearson r
0.50
0.25
0.00
−0.25
−log10 p−value
25
50
75
100

Figure S12

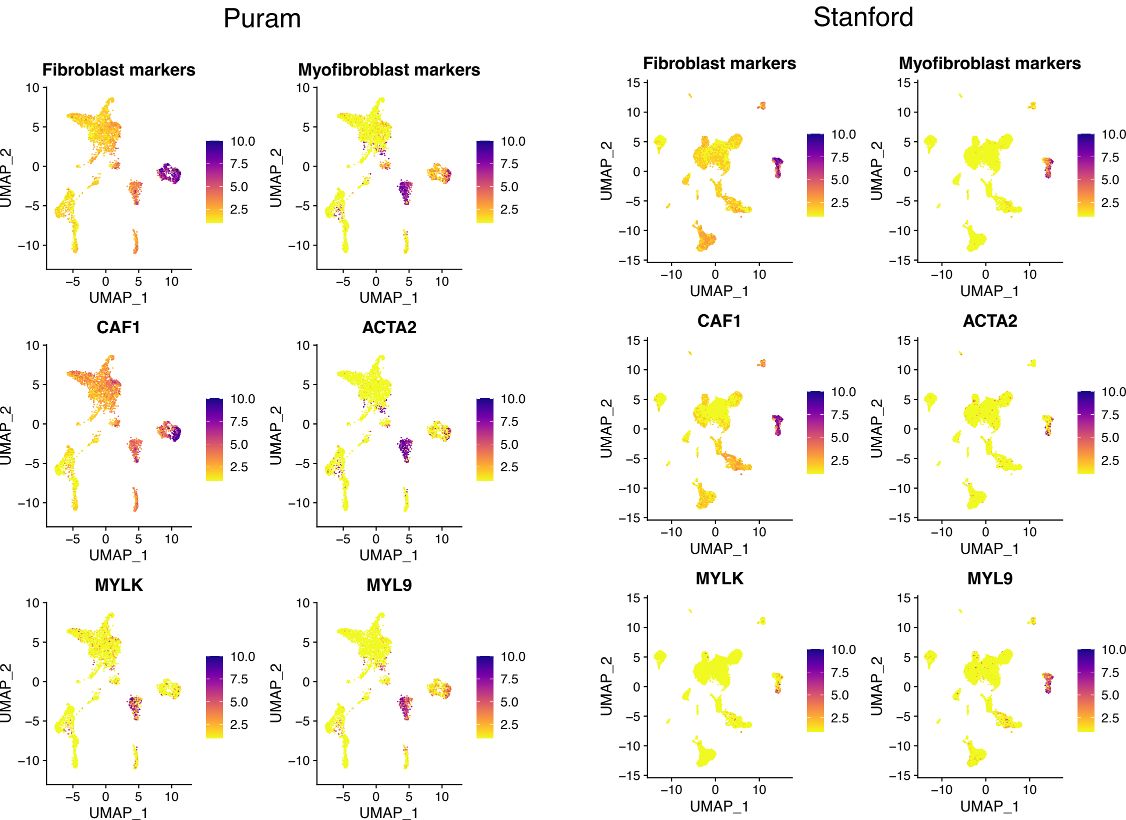

Puram
Stanford
Fibroblast markers
Myofibroblast markers
CAF1
ACTA2
MYLK
MYL9
UMAP_1
UMAP_2
10.0
7.5
5.0
2.5

Figure S13

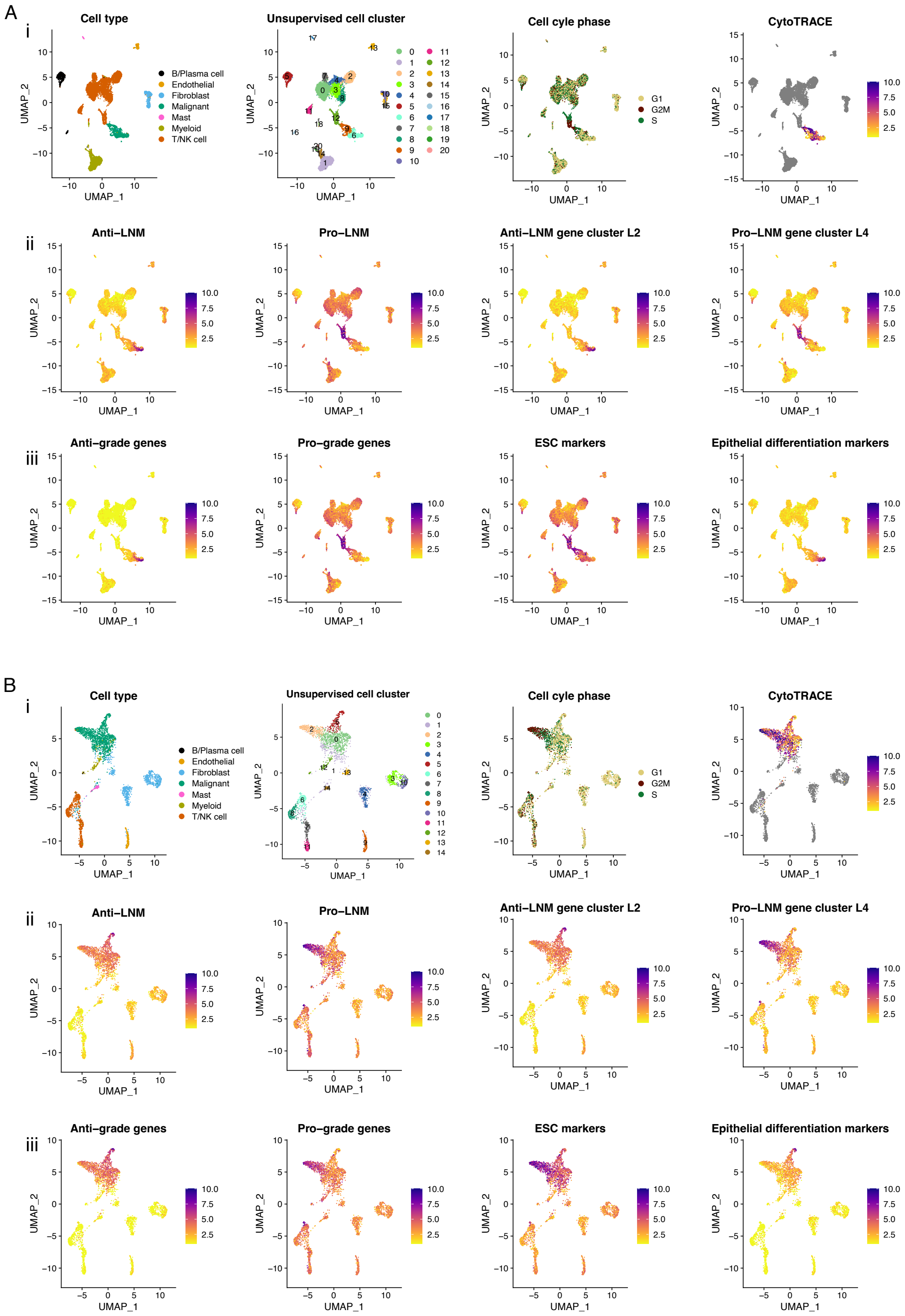
A
i
Cell type
B/Plasma cell
Endothelial
Fibroblast
Malignant
Mast
Myeloid
T/NK cell
Unsupervised cell cluster
Cell cyle phase
G1
G2M
S
CytoTRACE
ii
Anti–LNM
Pro–LNM
Anti–LNM gene cluster L2
Pro–LNM gene cluster L4
iii
Anti–grade genes
Pro–grade genes
ESC markers
Epithelial differentiation markers
UMAP_1
UMAP_2
B
i
Cell type
Unsupervised cell cluster
Cell cyle phase
CytoTRACE
ii
Anti–LNM
Pro–LNM
Anti–LNM gene cluster L2
Pro–LNM gene cluster L4
iii
Anti–grade genes
Pro–grade genes
ESC markers
Epithelial differentiation markers

Figure S14

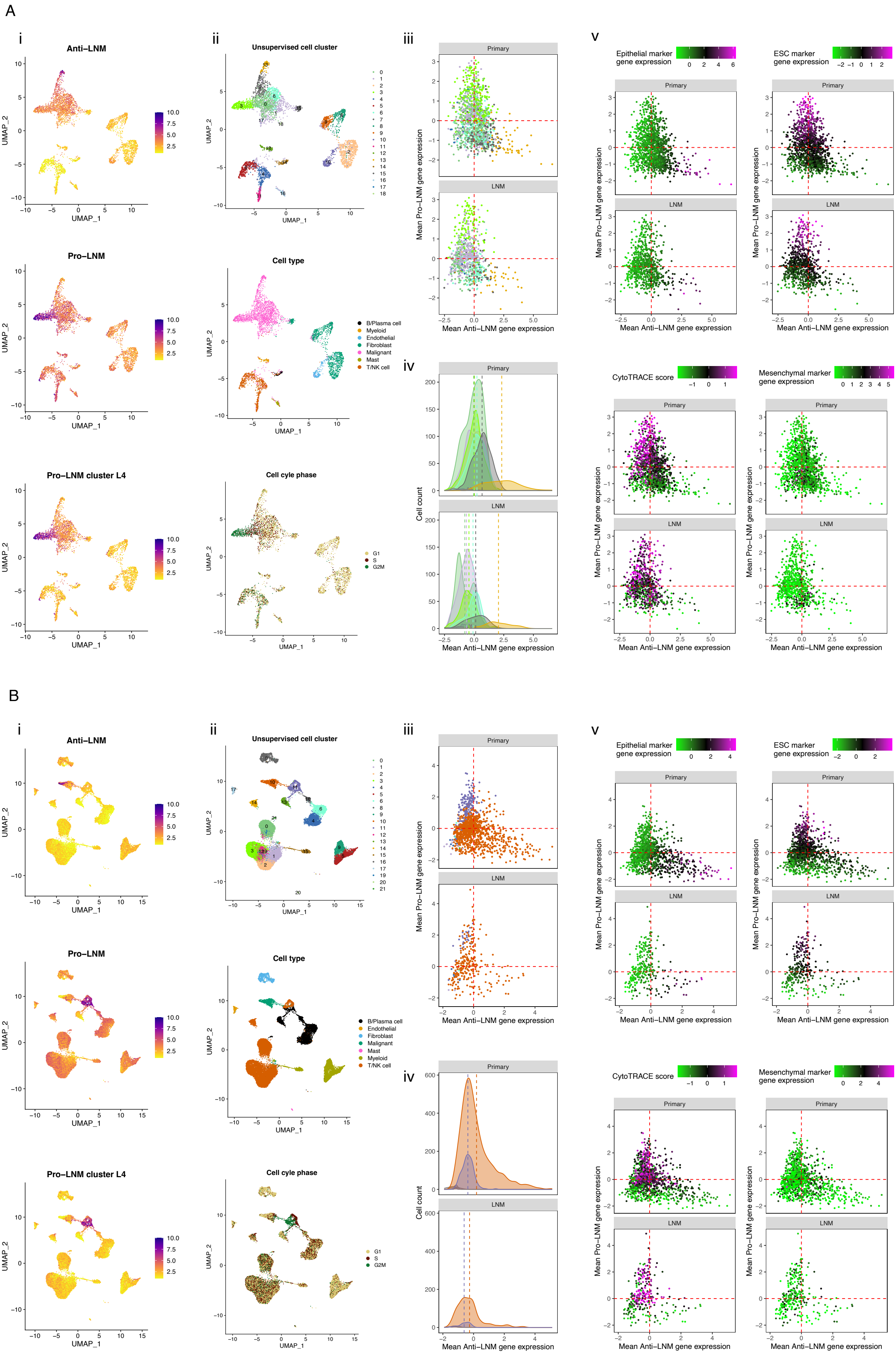

A
i
Anti–LNM
Pro–LNM
Pro–LNM cluster L4
UMAP_1
UMAP_2
ii
Unsupervised cell cluster
Cell type
B/Plasma cell
Myeloid
Endothelial
Fibroblast
Malignant
Mast
T/NK cell
Cell cyle phase
G1
S
G2M
iii
Primary
LNM
Mean Pro–LNM gene expression
Mean Anti–LNM gene expression
iv
Cell count
v
Epithelial marker gene expression
ESC marker gene expression
CytoTRACE score
Mesenchymal marker gene expression
B
i
Anti–LNM
Pro–LNM
Pro–LNM cluster L4
ii
Unsupervised cell cluster
Cell type
B/Plasma cell
Endothelial
Fibroblast
Malignant
Mast
Myeloid
T/NK cell
Cell cyle phase
G1
S
G2M
iii
iv
v
Epithelial marker gene expression
ESC marker gene expression
CytoTRACE score
Mesenchymal marker gene expression
